# Role of oceanic abiotic carbonate precipitation in future atmospheric CO_2_ regulation

**DOI:** 10.1038/s41598-022-20446-7

**Published:** 2022-09-24

**Authors:** Or M. Bialik, Guy Sisma-Ventura, Noam Vogt-Vincent, Jacob Silverman, Timor Katz

**Affiliations:** 1 Marine Geology and Seafloor Surveying, Department of Geosciences, University of Malta, Msida, Malta; 2 Israel Oceanographic and Limnological Research (IOLR), Haifa, Israel; 3 Department of Earth Sciences, University of Oxford, Oxford, UK; 4 Present Address: Dr. Moses Strauss Department of Marine Geosciences, University of Haifa, Haifa, Israel

**Keywords:** Carbon cycle, Marine chemistry, Carbon cycle

## Abstract

The oceans play a major role in the earth’s climate by regulating atmospheric CO_2_. While oceanic primary productivity and organic carbon burial sequesters CO_2_ from the atmosphere, precipitation of CaCO_3_ in the sea returns CO_2_ to the atmosphere. Abiotic CaCO_3_ precipitation in the form of aragonite is potentially an important feedback mechanism for the global carbon cycle, but this process has not been fully quantified. In a sediment-trap study conducted in the southeastern Mediterranean Sea, one of the fastest warming and most oligotrophic regions in the ocean, we quantify for the first time the flux of inorganic aragonite in the water column. We show that this process is kinetically induced by the warming of surface water and prolonged stratification resulting in a high aragonite saturation state (Ω_Ar_ ≥ 4). Based on these relations, we estimate that abiotic aragonite calcification may account for 15 ± 3% of the previously reported CO_2_ efflux from the sea surface to the atmosphere in the southeastern Mediterranean. Modelled predictions of sea surface temperature and Ω_Ar_ suggest that this process may weaken in the future ocean, resulting in increased alkalinity and buffering capacity of atmospheric CO_2_.

## Introduction

The production of CaCO_3_ minerals (namly, aragonite and calcite) plays an important role in regulating the ocean’s carbon budget^1,2^. Whereas most of the CaCO_3_ production in the oceans is biogenic, a fraction forms in abiotic reactions. To date, abiotic CaCO_3_ production in the form of aragonite has been observed in certain localities, such as the Bahamas or the Persian Gulf, where specific conditions are present. However, as ocean acidification intensifies, the production of abiotic CaCO_3_ will diminish and may have already diminished significantly, due to the reduction in the saturation state of the carbonate ion. Here we show, for the first time, the occurrence of abiotic aragonite production in the eastern Mediterranean, under different conditions than previously observed and a possible analogue to future ocean conditions under the effects of global warming. According to the proposed mechanism, the abiotic production of CaCO_3_ in surface waters may be enhanced by warming and stratification of the oceans, rather than additional seeding or water mass mixing. In contrast, ocean acidification may inhibit the abiotic production of surface waters, countering the positive effect of warming. Based on our observations, we argue that abiotic aragonite production is more ubiquitous than previously considered and delineate the production mechanism. Based on state-of-the-art climate models, we illustrate that the potential that this mechanism is diminishing and discuss its impact.

## Background

The exchange of CO_2_ between the ocean and the atmosphere has been considered to act as a major feedback mechanism that helps regulate planetary climate^1,2^. Currently, it is estimated that ca. 25% of anthropogenic CO_2_ emitted to the atmosphere annually is absorbed by the ocean^3,4^. The ensuing chemical reaction of the absorbed CO_2_ with seawater produces carbonic acid, resulting in a process known as ocean acidification^5^. The production of carbonic acid not only reduces seawater's pH, but also shifts the speciation of dissolved inorganic carbon (DIC = CO_2_ + HCO_3_^−^  + CO_3_^−2^) in seawater away from carbonate (CO_3_^−2^) to bi-carbonate (HCO_3_^−^), according to the following stoichiometric equation –  CO_2_ + H_2_O + CO_3_^−2^ → 2HCO_3_^−^.

The speciation of DIC in seawater is also influenced by temperature through its effect on the thermodynamic dissociation constants, where warming shifts the carbonate system towards CO_3_^−2^. Most of the transformation of DIC into solid phase, be it organic or inorganic, is biologically-driven^6^. Where, biologically mediated assimilation of DIC into inorganic carbon solid phase (CaCO_3_) removes Ca^+2^ from seawater and results also in a reduction of seawater total alkalinity (TA). This process is also non conservative with salinity changes^7^. While the organic sink (“biological pump”) removes carbon from the atmosphere, the inorganic sink (precipitation of carbonate minerals), consumes TA and shifts the carbonate system towards CO_2_ and its degassing from the ocean surface to the atmosphere^8^, acting as a positive feedback to global warming^2,9^. Precipitation of both crystal forms of CaCO_3_ (aragonite and calcite) in the ocean is mostly biogenic^10^ and is a highly complex process, affected by environmental conditions, such as temperature, dissolved nutrient levels and, most noteworthy, the ratio of the Ca^+2^ and CO_3_^−2^ ion activity product to the solubility constant of CaCO_3_, also known as the CaCO_3_ saturation state (Ω)^9^.

Oceanic surface waters are mostly supersaturated with respect to CaCO_3_ (Ω > 1) and therefore, one would rightfully expect the abiotic precipitation of CaCO_3_ to be a common occurrence and an important process in the oceanic carbon cycle and climate regulation. However, to date, large spatial scale abiotic CaCO_3_ precipitation (usually aragonite) in the marine environment have been observed only during events referred to as “whitening events”, when CaCO_3_ is abiotically precipitated in surface waters, turning them milky white in relatively shallow waters^11–13^. Such events occur almost exclusively in the Persian Gulf and the Bahamas. While the specific mechanism driving whitening events is still a highly contested topic^14–16^, extremely high levels of Ω seem to be an important factor triggering them.

The abiotic nucleation of CaCO_3_ in seawater is strongly inhibited kinetically^17,18^. Suspended particles, which provide a mineral surface area for nucleation (“seeding”), were also suggested to play an important control for carbonate precipitation from seawater^16,19^. However, “seeded” experiments have concluded that temperature controls the mineralogy of the precipitating phase and that the precipitation rate is proportional to the saturation state^20,21^. Hence, vast low latitude oceanic regions, where the saturation state is high^22^ and most CO_2_ degassing occurs^23^, are suspected to accommodate abiotic precipitation of aragonite. Yet, this process remains largely undetected, as it is often concealed by the larger biological signal^8,9^.

### Abiotic aragonite precipitation

As the ocean warms and stratification intensifies, primary productivity decreases, most notably in oligotrophic regions^25,26^. This would mean that the carbon cycle in these regions may sway from biological mediation towards greater abiotic (chemical and physical) control. This process is already ongoing in the southeastern Mediterranean^27,28^ making it a good model system for the warmer stratified future of the oceans. Like most oceanic basins, the region is experiencing acidification, yet, the rate of acidification is relatively low due to increasing alkalinity (supplemental 1, Fig. S1.1). In contrast, the summertime sea surface temperature has risen at an exceptionally high rate of up to 1 °C/decade over the last three decades^29,30^, substantially higher than warming rates in other low latitude tropical seas^31^. Additionally, the oligotrophic state of the region results in one of the lowest primary productivities of any marine water body in the world^32^.

**Figure 1:**
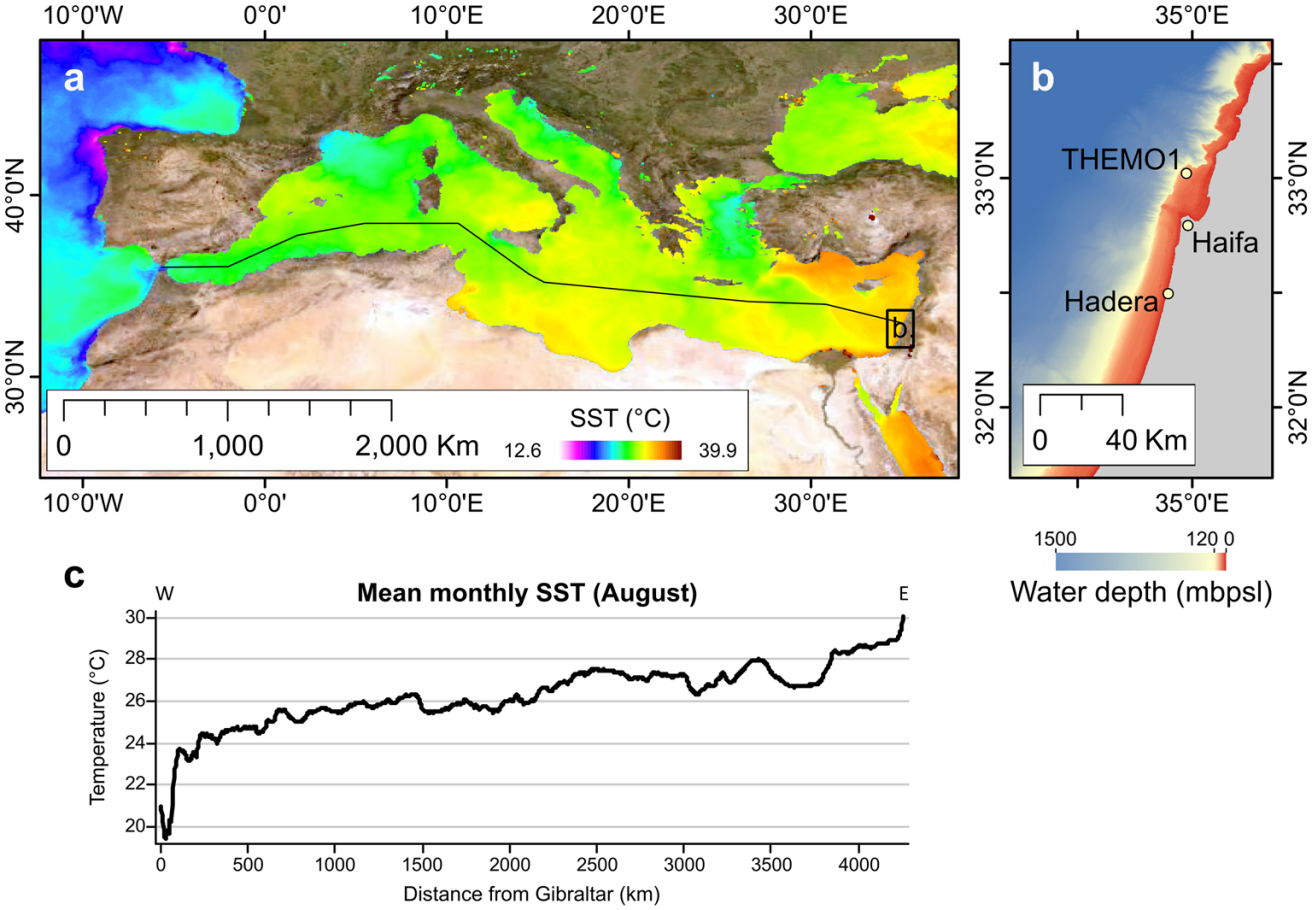
(**a**) Location map of the Mediterranean Sea showing sea surface temperature (SST) in °C for August 2016, Data from MODIS|Aqua (https://oceancolor.gsfc.nasa.gov/). (**b**) bathymetric map of the Israeli shelf^24^ showing the location of the Hadera and THEMO1 stations. (**c**) SST temperature prolife along the transect shown in (**a**).

**Figure 2:**
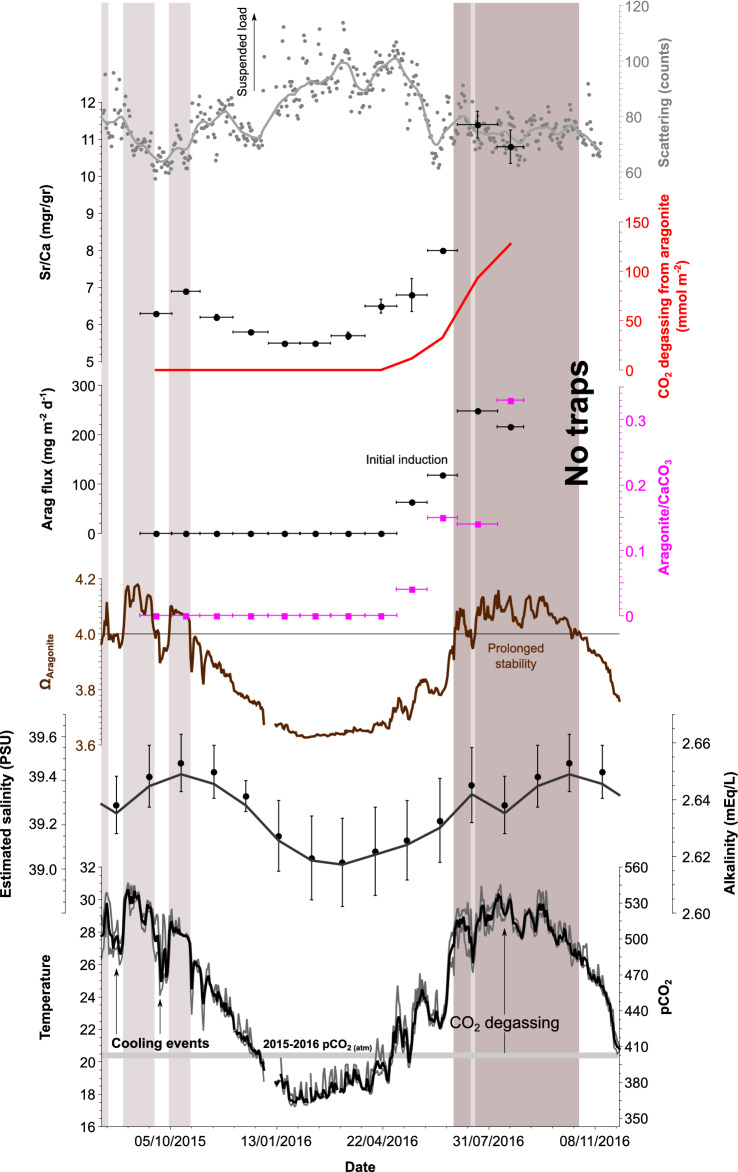
Record from the Hadera station showing (from the bottom), temperature (at 26 m) and inferred pCO_2_, mean monthly salinity for the given month and inferred alkalinity, calculated saturation index for aragonite, aragonite fraction of total carbonate, flux of aragonite to the trap, inferred total CO_2_ degassing from aragonite precipitation, Sr/Ca ratio of the sediment in the trap, and acoustic backscatter. Shaded areas indicate periods when seawater Ω_Ar_ > 4 (see Fig. 3).

In a sediment-trap study that we conducted in the southeastern Mediterranean Sea (Fig. 1), we quantify for the first time the flux of inorganic precipitation of aragonite in the water column during the summer (supplemental 2). Unlike calcite, there are only a few sources of biogenic aragonite in the study area, e.g. pteropod blooms and resuspension of eroded aragonite shells of benthic organisms – none of which were present in the traps based on microscopic examination of the material in the traps and adjacent surface sediments. The Sr/Ca ratios in the traps ranged between 6.8 and 11.4 in the summer months when aragonite was present (Fig. 2; supplemental 1, Table S1.1). The Sr/Ca ratio in the sediment was found to be ~ 5.0 and biogenic sources such as pteropods were found to have ratios ranging between 2.3 and 3.6 (supplement 1, Table S1.2). As a result, neither of these sources can explain the higher Sr/Ca ratio in the traps. However, these values agree well with the Sr/Ca ratio (~ 9.0) of abiotic aragonite precipitated in lab experiments from seawater in the range of summertime temperatures in the study area^33^. This is still lower than the maximum value of Sr/Ca = 11.4, where the excess Sr is likely sourced from Saharan dust, which has a Sr/Ca ratio of ~ 30^34,35^. However, the dust is aragonite-free and its supply rate is low during summer^36^. Thus, neither dust nor resuspension, given the low wave activity during summer, explain the relatively high Sr/Ca ratio in the traps. Thus, it is reasonable to conclude that it is caused by abiotic precipitation of aragonite (supplemental 1, Figs. S1.2–S1.3).

Recently, it was suggested that abiotic aragonite precipitation may be caused by seeding, where aragonite crystallizes on suspended nucleation particles^37,38^. In this model, a high load of nucleation seeds/surface area promotes increased precipitation^9^. The saturation state, while elevated (≥ 4), does not appear to be sufficient for spontaneous nucleation of aragonite from seawater^39^. Therefore, our results support utilization of preexisting nuclei in the water mass by a sustained high aragonite saturation state during summer. Acoustic backscatter data from the ADCP adjacent to the sediment trap (Fig. 2), a proxy for turbidity in the water, indicated highest turbidity between January and May, and much lower turbidity during the summer months. This trend was mirrored in the total mass and CaCO_3_ fluxes in the sediment traps (supplement 1; Table S1.1) decreased from 82 and 15.8 gr m^−2^ d^−1^ in January 2016 to 2.9 and 0.7 gr m^−2^ d^−1^ in August 2016, respectively. As such, we would argue that even in stratified, low-turbidity environments, precipitation nuclei are not a limiting factor for inorganic precipitation of aragonite.

**Figure 3:**
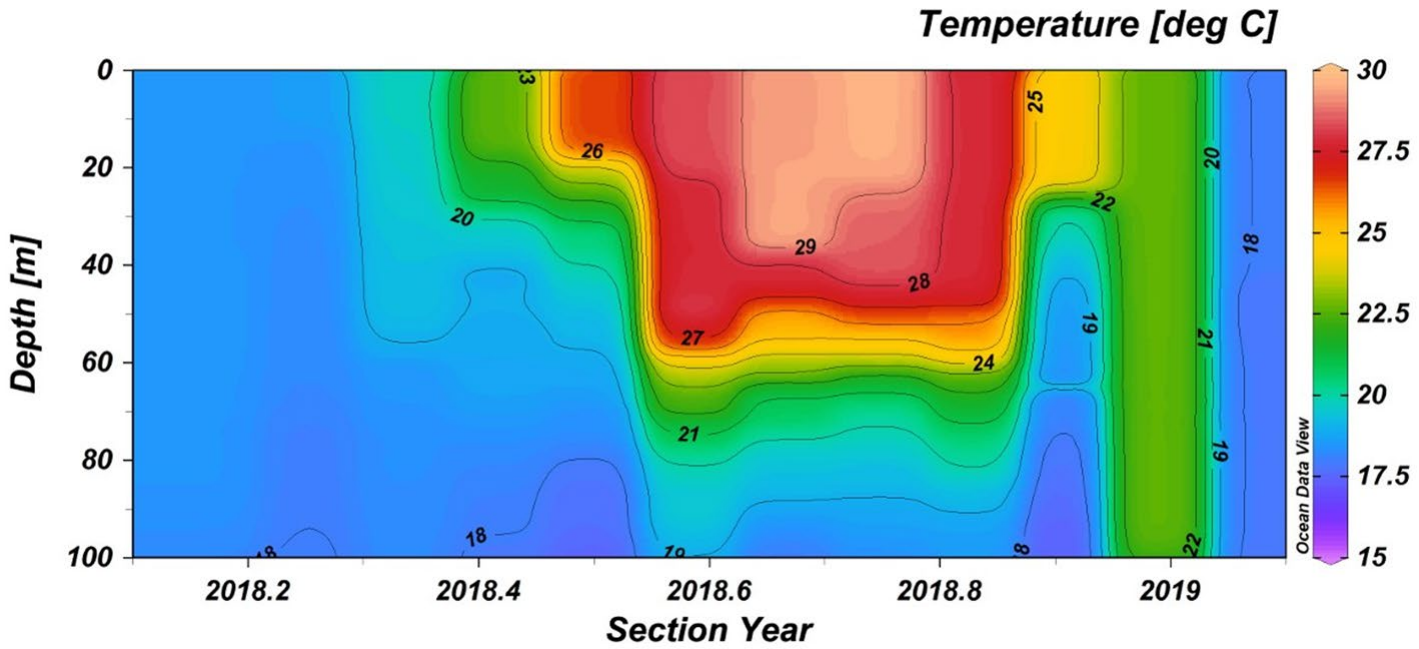
Time series of temperature in the upper 100 m of the water column at THEMO-1 station (early 2018 to early 2019) showing the summertime stratification of the water column.

**Figure 4:**
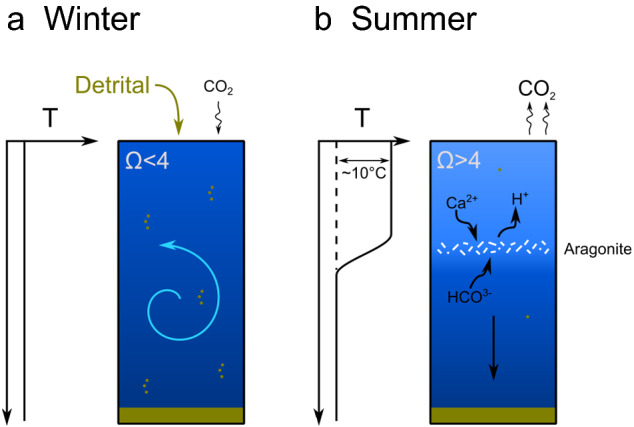
Schematic model for aragonite formation in the Eastern Mediterranean. During cooling events and vertical mixing of the water column (**a**), the cooler water with increased productivity acts as a CO_2_ sink. During prolonged periods of warming (**b**), the water column becomes thermally stratified, CO_2_ degasses due to super saturation, CO_3_^−2^ and Ω_Ar_ increase, and abiotic aragonite precipitation occurs and intensify CO_2_ supersaturation and degassing.

The other major factor for the precipitation of aragonite is the seawater CaCO_3_ saturation state. Abiotic nucleation of aragonite has been demonstrated to be possible with Ω_Ar_ ≈ 4, given induction time (the period of time that is necessary to initiate a reaction) of around two months^37^. To maintain stable Ω_Ar_ conditions, a prolonged period of water column stability is required which is consistent with the stratification of the surface water. Our observations show that aragonite formation started in late-spring and continued throughout the summer months, between May and October 2016 (Fig. 2), corresponding to the time of year when warming and weaker mechanical mixing causes intense stratification of the surface layer (Fig. 3). At its apex, the gradient across the seasonal thermocline was on the order of 10 °C (supplement 1; Fig. S1.4). As this layer concentrates heat at the surface, pCO_2_ levels reach super saturation in excess of 450 μatm (Fig. 2). During winter, the upper water column is mixed (Fig. 3), temperature and Ω_Ar_ are lower (Fig. 2), while biogenic production and sediment load are higher, suppressing abiotic formation of aragonite (Fig. 4a). The absence of abiotic aragonite in the sediment traps during the autumn of 2015 when Ω_Ar_ was at times > 4, is likely the result of two notable (4–6 °C) cooling events that occurred in August and September 2015. These events lasted several days (Fig. 2) and broke the stratification, resetting the required induction time of Ω_Ar_. Notably, 2015 was an anomalous year with the least number of days with Ω_Ar_ > 4 in the last decade (supplemental 1, Fig. S1.5). Additionally, while nearly constant Ω_Ar_ > 4 conditions in local surface waters prevail throughout the summertime, when stratification is most intense (supplement 1, Fig. S1.6), when that stratification is broken, as in winter (supplement 1, Fig. S1.7), values of Ω_Ar_ drop below 4. The aragonite found in the traps in late May to early June 2016 likely had formed higher in the water column as the stratification and super-saturation conditions began already in early April at shallower depths and extended into the summer^28^ (Fig. 3), allowing for aragonite formation (Fig. 4b).

The estimated flux of aragonite based on the trap data from 2016 would be ~ 0.20 ± 0.04 mol m^−2^ year^−1^ for the deployment period. Assuming that this is a representative flux of inorganic carbonate production, this would correspond to a CO_2_ efflux^8^ of 0.12 ± 0.01 mol CO_2_ m^−2^ year^−1 ^(supplemental 4). The total annual net CO_2_ flux to the atmosphere in the southeastern Mediterranean Sea was previously estimated at 0.85 ± 0.27 mol m^−2^^28^. This suggests that the abiotic aragonite precipitation may account for ~ 15 ± 3% of the annual net CO_2_ flux from the sea surface to the atmosphere in the southeastern Mediterranean. Relatively stable and consistent sea surface temperature and salinity conditions in the region^40^ allow for the assumption that this represents at least the region of the Levant Basin and possibly extends beyond it in the southeastern Mediterranean. This net annual calcification flux per unit area is only ~ 0.5% of CaCO_3_ flux estimated for coral reefs^41^, but the potential area of production is orders of magnitude larger, making it a significant source of atmospheric CO_2_.

This is the first time that the relation between warming, stratification, abiotic aragonite precipitation and CO_2_ release is demonstrated in any marine system. The observed link between surface warming and CO_2_ efflux due to abiotic aragonite precipitation provide a new mechanism by which warming reduces the southeastern Mediterranean Sea buffering capacity for sequestering atmospheric CO_2_ (and possibly even becoming a CO_2_ source^28^). This is a novel feedback mechanism of the carbonate system under global warming.

### Implications

**Figure 5:**
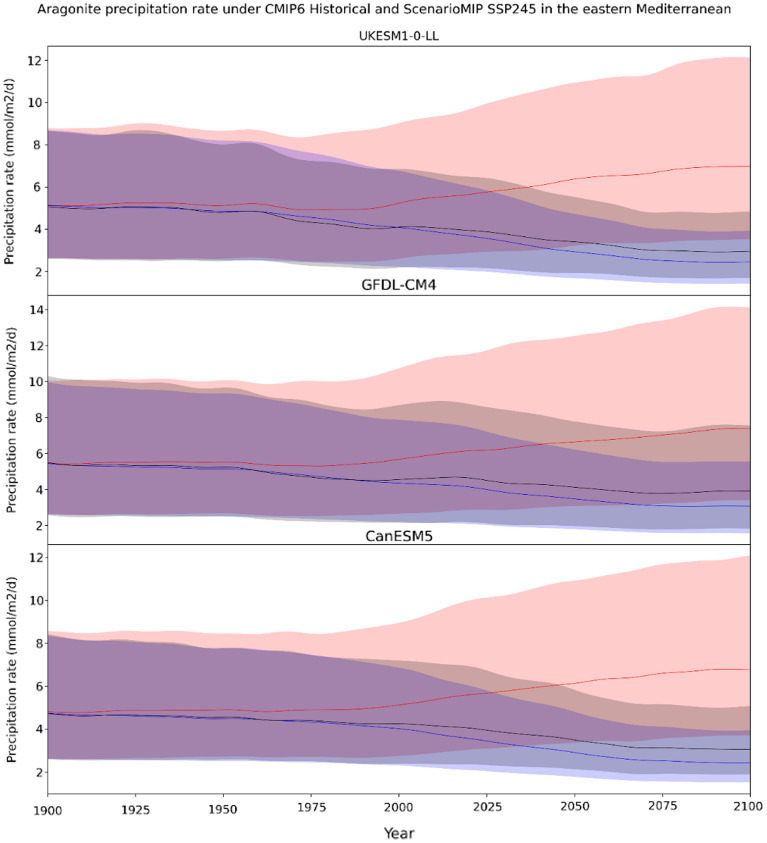
The change in $R_{ai}$ (abiotic aragonite precipitation rate) over time from model simulations. $R_{ai}$ (black line), $R_{ai}^{SST - only}$(red line) and $R_{ai}^{\Omega - only}$(blue line) from CMIP6 Historical and ScenarioMIP SSP2-4.5 for the eastern Mediterranean. The $R_{ai}$ scenario reflects the ‘true’ CMIP prediction, whereas the $R_{ai}^{SST - only}$ and $R_{ai}^{\Omega - only}$ scenarios fix $\Omega_{Ar}$ and SST, respectively to 1850–1899 climatological values. The top, central and bottom panels are UKESM1-0-LL, GFDL-CM4, and CanESM5, respectively. Shaded area indicate the range of error while the lines represents the annual mean (of monthly mean $R_{ai}$ calculated across the eastern Mediterranean) and the shaded region spans the annual range.

More broadly, it was recently shown that much of the lower latitude surface ocean is characterized by Ω_Ar_ ≥ 4, and that Ω_Ar_ is positively correlated with temperature^22,42,43^. As the lower latitude ocean warms, much of that heat is stored in the surface waters, resulting in enhanced stratification and oligotrophy^25,26,31^. These three properties (warming, stratification and oligotrophy) will likely modify the balance of the carbon system, thereby affecting the ocean’s capacity to store CO_2_^44^. Warming, in particular, is of significant importance as it affects the apparent rate of CaCO_3_ precipitation through the kinetic rate coefficient K_T_ and order of reaction n_T_ (which are temperature dependent; supplement 3; Fig. S3.1). Thus, if the temperature increases K_T_ and n_T_ faster than ocean acidification decreases Ω_Ar_, the net apparent CaCO_3_ precipitation rate may increase. However, atmospheric buildup of CO_2_ is faster than warming in terms of their combined and individual effects on K_T_ and Ω_Ar_ (supplement 3). Under the influence of warming alone, global potential aragonite production rate would increase, but due to the effect of CO_2_ induced acidification, they have been and are expected to continue to drop (Fig. 5). As a result, the abiotic aragonite sink may be weakening, resulting in reduced TA uptake and non-conservative build-up of TA in the oceans as suggested by the Hawaiian Ocean Time Series salinity normalized TA time series (supplement 1, Fig. S1.8). In turn, this TA build-up could potentially increase the ocean’s buffering capacity for increasing atmospheric CO_2_, increasing oceanic uptake. However, it should be noted that decadal changes in terrestrial runoff, deep mixing and evaporation regimes in and around the Pacific could also explain the non-conservative long-term trend in salinity^45^. Regardless, the exact contribution of abiotic aragonite precipitation in the upper water column cannot be fully quantified at this time—but it is a feedback mechanism that should be further investigated and incorporated into planetary models to move towards a more complete description of the ocean/atmosphere system.

In the geological record, shifts between periods of aridity and strong oceanic stratification with high aragonite/calcite ratios and periods of high humidity, cooler mixed water column conditions with low aragonite/calcite ratios, are evident in low latitude sediment cores^42^. Taking into account that surface seawater pCO_2_ is mostly temperature-dependent, and that ocean temperatures will continue to increase in the near future^46^, a reduction in the ocean’s capacity to absorb CO_2_ is to be expected under prolonged stratification. In both Earth’s past and near future, warming due to the earth orbital changes could enhance the abiotic carbonate formation, potentially resulting in a greater release of CO_2_ from the surface waters. The switch from cooler waters (glacial/icehouse periods), where carbon is removed by the biological pump to warm (interglacial/greenhouse period), stratified and oligotrophic surface water, where abiotic aragonite is precipitated may have altered the oceanic buffering capacity. This means that the surface water will become a much less effective sink and under extreme scenarios, larger areas of the surface ocean may become a CO_2_ source. However, our proposed feedback mechanism suggests a future reduction in abiotic aragonite precipitation under the increasing effect of ocean acidification. The unprecedented rate of anthropogenic climate change is not paired with a comparable increase in continental weathering. In the geological past,  weathering may have provided TA to sustain this mechanism, at least in epicontinental seas which could decouple with respect to Ca^2+^ and Mg^2+^ from the ocean^47^.

We therefore suggest that the feedback between warming, acidification, and induced CO_2_ release due to abiotic aragonite precipitation can be regarded as a potential feedback to global warming, adding to a growing list of feedbacks such as reduced primary productivity in the ocean, desertification, and melting of permafrost^46^, and therefore should be accounted for in future estimation of ocean evolution in response to climate change.

### Methods

Samples were collected from sediment traps deployed 2 km offshore of Hadera, Israel at 25 m bottom depth. Analysis of sediment samples was carried out using X-ray fluorescence (XRF) and X-ray diffraction (XRD). Seawater carbonate chemistry parameters were estimated from in situ measurements of temperature and salinity from the ISRAMR data base, which were converted to pCO_2_ and total alkalinity respectively. See supplement 2 for more information on methods. Global state of the carbon system was estimated from CMIP6 products, see supplement 3 for more information.

## Supplementary Information

Supplementary Information 1.

Supplementary Information 2.

Supplementary Information 3.

Supplementary Information 4.

## Data Availability

Scripts, results and additional material for model simulations are available via a the Figshare repository at 10.6084/m9.figshare.15121131; all other material in the supplement are available at 10.6084/m9.figshare.1972181. Oceanographic data used in this manuscript is available through the ISRAMAR (https://isramar.ocean.org.il/isramar2009/) databases. see https://themo.haifa.ac.il/ for more information on the THEMO project. Additional notes on data are listed in supplements 2 and 3.
