# Supplementary material for: Role of oceanic abiotic carbonate precipitation in future atmospheric CO2 regulation
Source: Sci Rep. 2022 Sep 24;12:15970. doi: 10.1038/s41598-022-20446-7 (PMC9509385; doi:10.1038/s41598-022-20446-7)
Supplement: Supplementary file 1 — Supplementary Information 1. [file 41598_2022_20446_MOESM1_ESM.docx]

Supplement 1 - ongoing trends in the Mediterranean and global ocean

**Figure S1.1**: long term pH and alkalinity record for the Levant region (Bialik & Sisma-Ventura, 2016; Sisma-Ventura et al., 2016, 2017; THEMO record). The records demonstrate an ongoing increase in alkalinity related to increase in salinity (evaporation + damming of the Nile river) and an ongoing acidification trend from the 1970s to the early 2000s followed by stabilization (discussed partially in; Sisma-Ventura et al., 2017). Note that some of the variability in the data probably stems from different measurements methods.


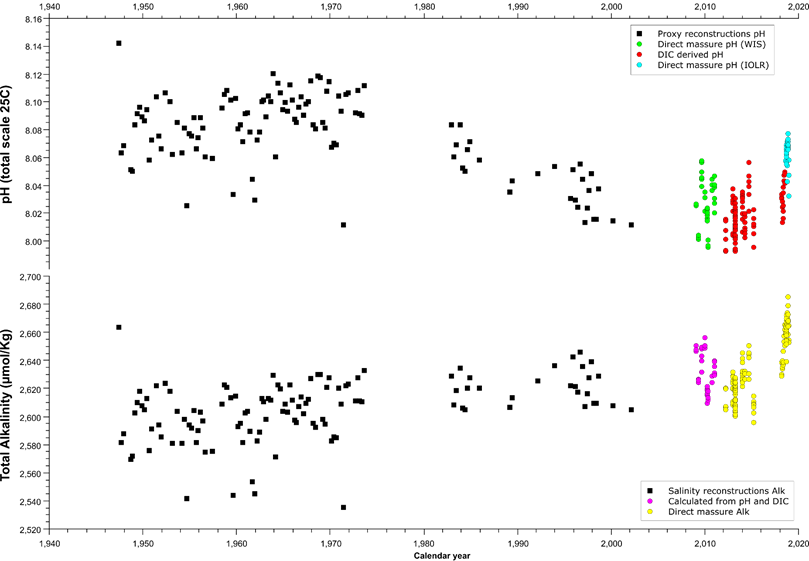


**Figure S1.2**: Wave conditions (mean period and max wave height) in Hadera during the deployment period, note that during summer wave activity diminishes significantly with 2016 being calmer than 2015. Data provided by IOLR.

**Figure S1.3:** Available data for wave conditions (mean period) in Hadera during September 2015 and 2016.

**
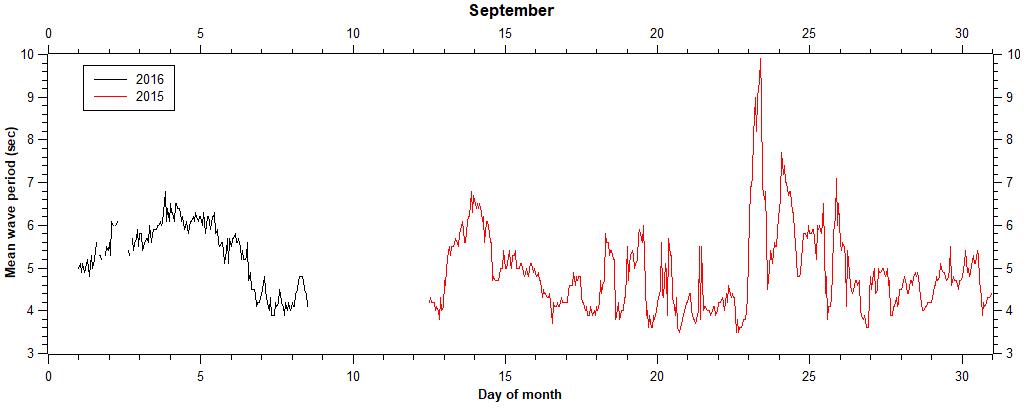
**

**Figure S1.4**: Salinity and temperature profiles for a. February 2018 and b. August 2018 from the THEMO 1 station representing the two-end member of stratification at the SEMS. During winter (February) the upper 100m are well mixed where as in summer (August) three distinct intervals form.


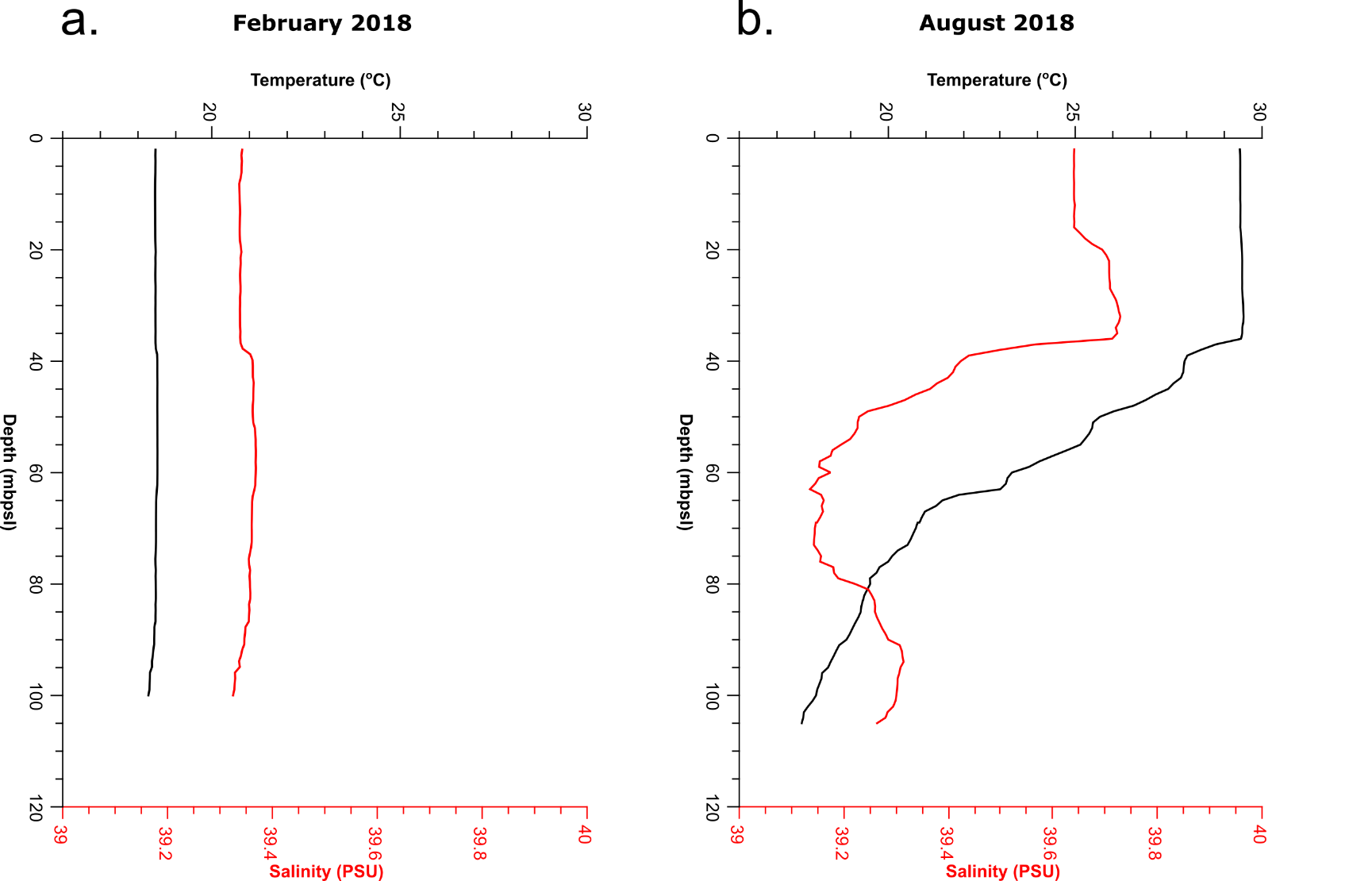


**Figure S1.5**: Number of days per year (2004-2018) with Ω_Aragonite_>4 calculated to the Hadera station, horizontal line mark mean (black) and standard diviations (grey). Note that 2007, 2010, 2013 and 2015, which had relatively cool summer (Pastor et al., 2020) are anomalous. Ω_Aragonite_ was calculated using the background multiannual salinity values alkalinity estimation and pCO_2_ calculated from in situ temperature measurements (following Sisma-Ventura et al., 2017; supplument 2). Daily Ω_Aragonite_ were averaged and total days per years were sum per year.


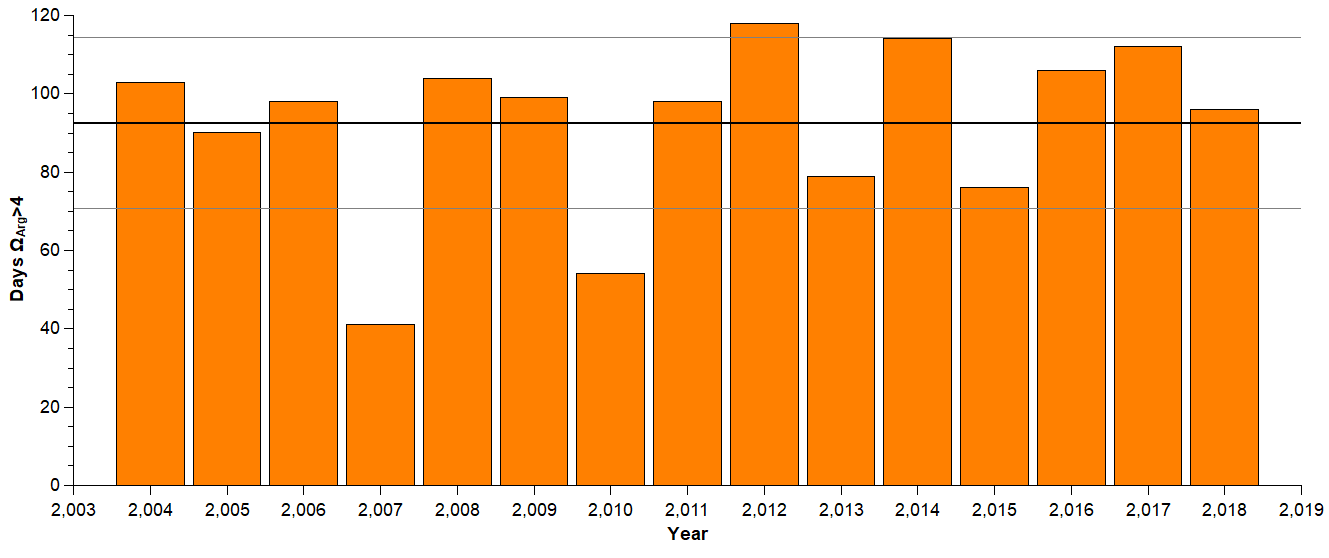


**Figure S1.6**: values of Ω_Aragonite_ in the surface waters during August 2019 along the Israeli shore, nearshore and open waters. Note that in every sample Ω_Aragonite_>4. Data collected and provided by the Israeli national monitoring program operated by IOLR.


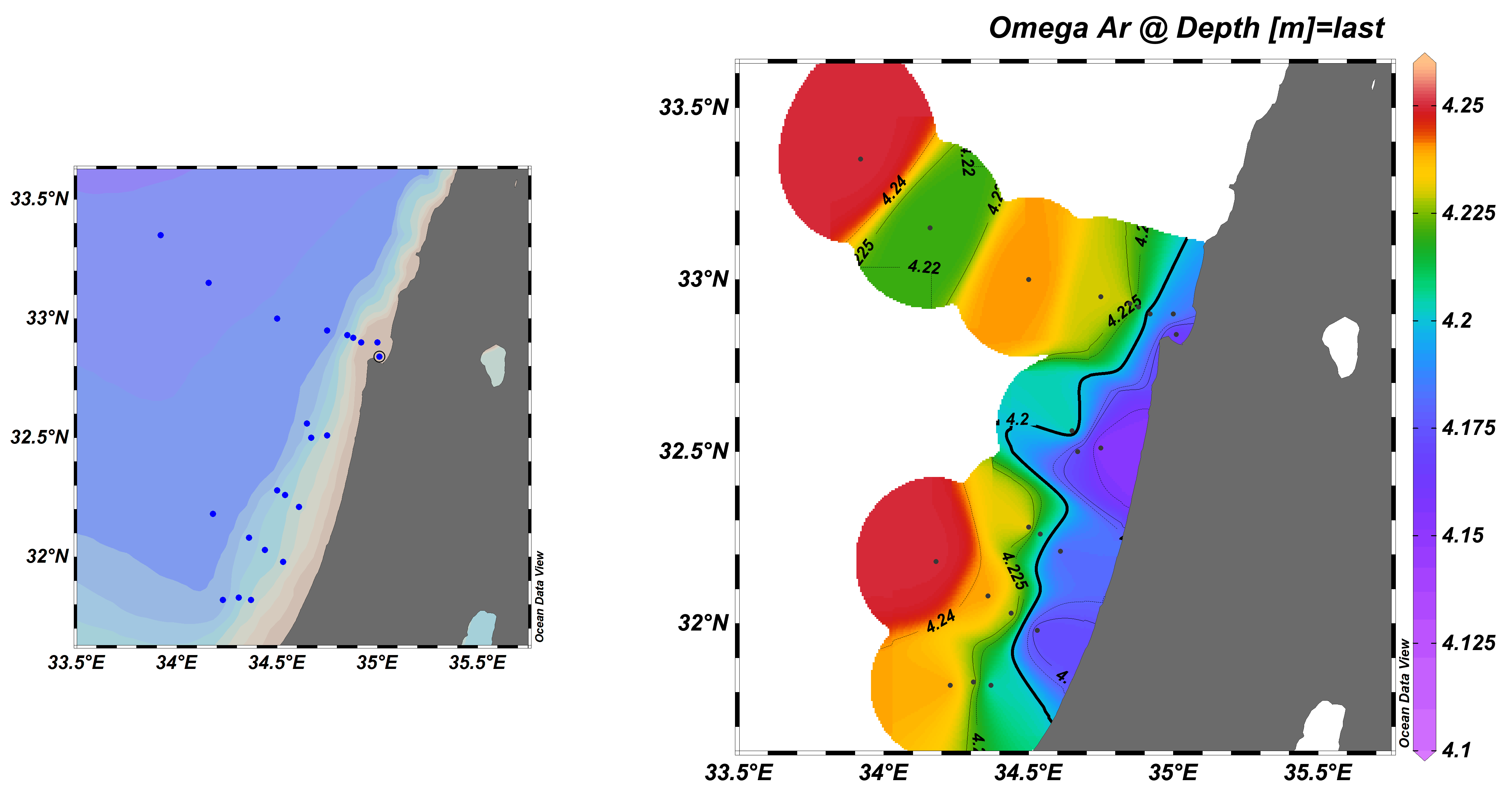


**Figure S1.7**: values of Ω_Aragonite_ along a shore to open waters transect from Haifa bay (north of Hadera) during summer 2019 in the period during which the water column is mixed. Ω_Aragonite_ diminishes to below 4 during the period the stratification is broken. Data collected and provided by the Israeli national monitoring program operated by IOLR.

**
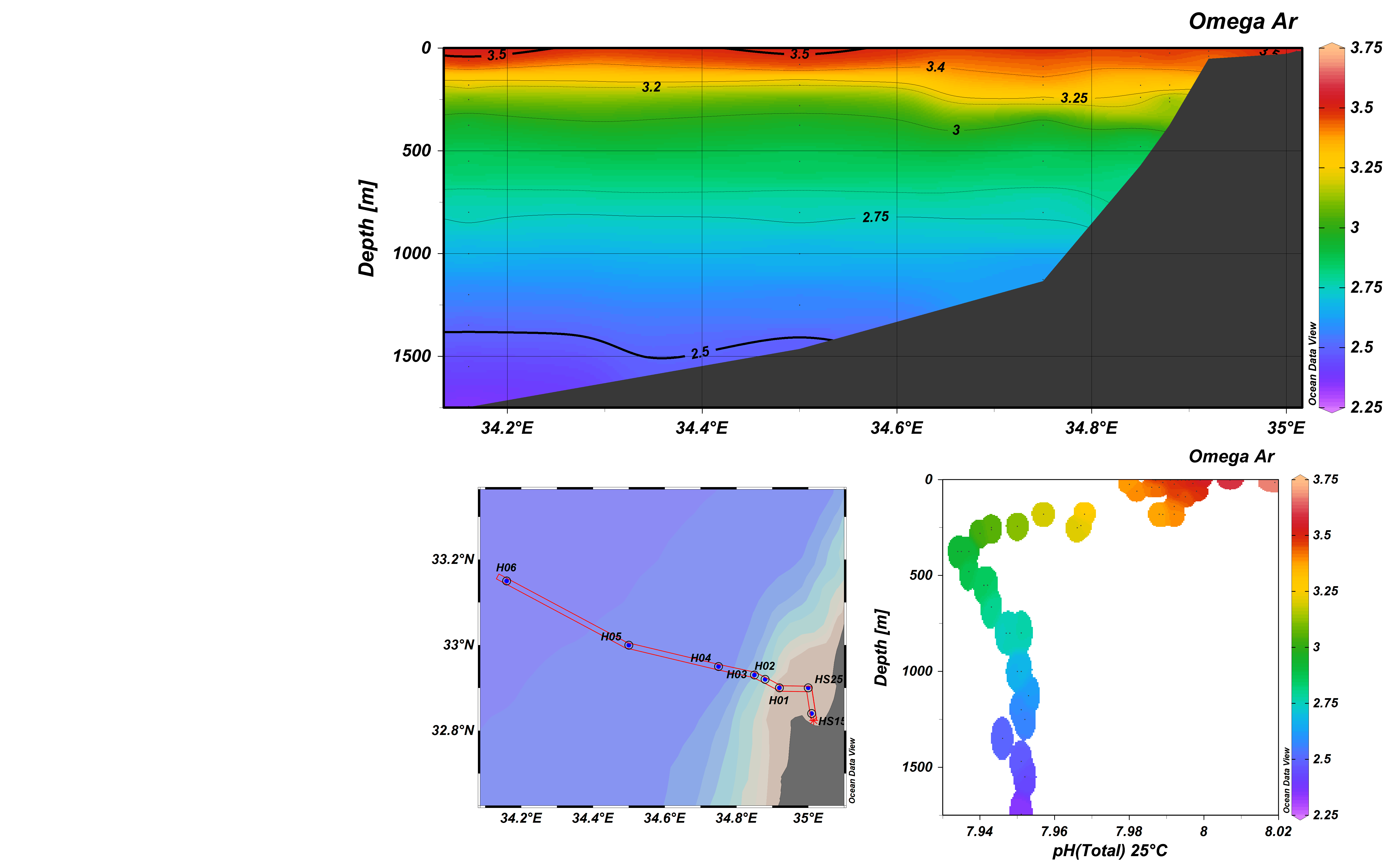
**

**Figure S1.8**: Salinity normalized total alkalinity in the surface water of the HOT station (<https://hahana.soest.hawaii.edu/hot/>) from 1988 to 2019. A +2 µmol/kg/decade positive trend of excess alkalinity is observed over the recorder period. This increase points to accumulation of alkalinity in the oceans.


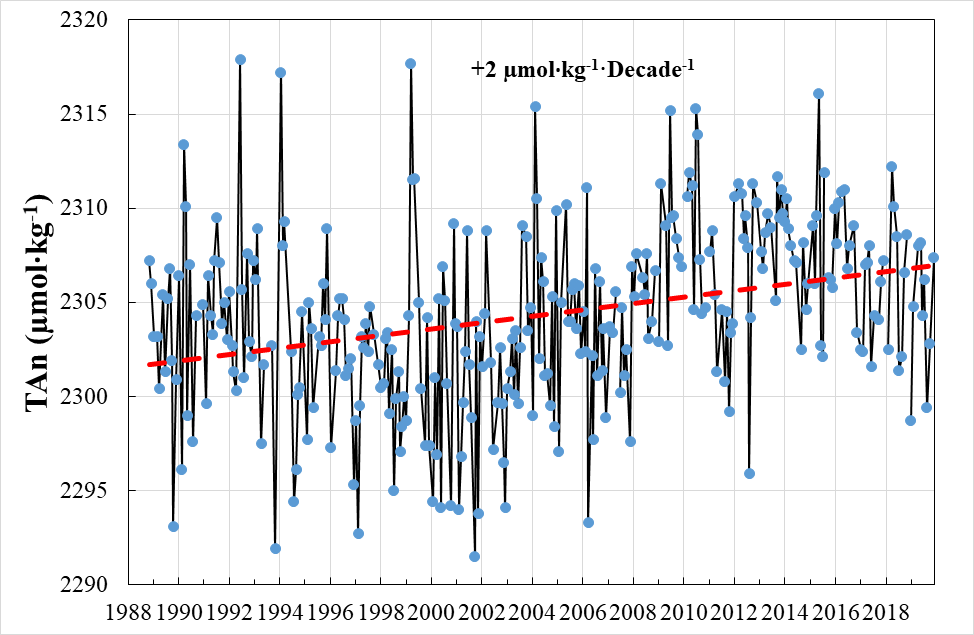


| **Middle term** | **27.9.15**  **(30)** | **25.10.15**  **(27)** | **23.11.15**  **(31)** | **25.12.15**  **(33)** | **26.1.16**  **(30)** | **24.2.16**  **(29)** | **26.3.16**  **(32)** | **26.4.16**  **(30)** | **25.5.16**  **(29)** | **23.6.16**  **(28)** | **26.7.16**  **(38)** | **26.8.16**  **(25)** | **Annual**  **(362)** |
| --- | --- | --- | --- | --- | --- | --- | --- | --- | --- | --- | --- | --- | --- |
| Aragonite/ CaCO_3_ (mean) | 0.00 | 0.00 | 0.00 | 0.00 | 0.00 | 0.00 | 0.00 | 0.00 | 0.04±0.06 | 0.15±0.00 | 0.14±0.02 | 0.33±0.14 | 0.05±0.04 |
| CaCO_3_ in traps (%wt) | 21.8 | 14.5 | 14.3 | 19.6 | 19.2 | 17.1 | 17.1 | 17.4 | 19.5 | 24.6 | 24.7 | 23.0 | 19.4 |
| TMF  g m^-2^ d^-1^ | 1.8 | 6.6 | 10.5 | 35.6 | 82.4 | 29.9 | 27.2 | 6.1 | 8.3 | 3.1 | 7.4 | 2.9 | 6807.8 |
| CaCO3 flux g m^-2^ d^-1^ | 0.4 | 1.0 | 1.5 | 7.0 | 15.8 | 5.1 | 4.6 | 1.1 | 1.6 | 0.8 | 1.8 | 0.7 | 1273.0 |
| Aragonite flux g m^-2^ d^-1^ | 0.0 | 0.0 | 0.0 | 0.0 | 0.0 | 0.0 | 0.0 | 0.0 | 0.1 | 0.1 | 0.2 | 0.2 | 19.9 |
| Sr/Ca (wt/wt) *1000 (mean) | 6.3±0.0 | 6.9±0.1 | 6.2±0.2 | 5.8±0.0 | 5.5±0.0 | 5.5±0.0 | 5.7±0.1 | 6.5±0.2 | 6.8±0.5 | 8.0±0.0 | 11.4±0.4 | 10.8±0.5 | 7.1±2 |
| Mean temperature (26 m, °C) | 27.6±1.8 | 26.3±1.5 | 23.6±0.9 | 20.8±0.8 | 18.1±0.6 | 18.1±0.2 | 18.7±0.4 | 20.3±0.6 | 22.6±1.4 | 25.5±1.8 | 28.8±1.0 | 28.9±0.7 | 23.3±4.7 |
| Salinity (multiannual) | 39.4±0.1 | 39.5±0.1 | 39.4±0.1 | 39.3±0.1 | 39.15±0.2 | 39.1±0.2 | 39.0±0.2 | 39.1±0.2 | 39.1±0.2 | 39.2±0.2 | 39.4±0.2 | 39.3±0.1 | 39.3±0.2 |
| Aragonite flux mmol m^-2^ d^-1^ | 0.0 | 0.0 | 0.0 | 0.0 | 0.0 | 0.0 | 0.0 | 0.0 | 0.6±0.02 | 1.2±0.00 | 2.5±0.01 | 2.2±0.03 | 6.4±0.04 |
| CO_2_ emission due to Aragonite mmol m^-2^ d^-1^ | 0.0 | 0.0 | 0.0 | 0.0 | 0.0 | 0.0 | 0.0 | 0.0 | 0.4±0.01 | 0.8±0.00 | 1.6±0.01 | 1.4±0.02 | 127.6±0.02 |
| Precipitated Aragonite per deployment (g m^-2^) | 0.0 | 0.0 | 0.0 | 0.0 | 0.0 | 0.0 | 0.0 | 0.0 | 1.8±0.1 | 3.3±0.0 | 9.4±0.0 | 5.4±0.1 | 19.9±0.1 |

**Table S1.1**: summary of data from sediment traps, deployment time as days in parenthesis. Note that the annual column lists total for fluxes and mean values for other properties. TMF stands for total mass flux. %CaCO_3_ is based on Ca measurements (see supplement 2).

| **Material** | **Sr/Ca ratio (**mg/g) | **Source** |
| --- | --- | --- |
| Pteropods shells | 3.60±0.03 | (Rutten et al., 2000) |
|  | 2.3 | This study |
| Glycymeris shells | 7 | This study |
| Local sediment (Hadera) | 5.0±0.1 | This study |
| Saharan dust | 28.4±1.4 | (Krom et al., 1999) |
|  | 28.7±1.2 | (Rahav et al., 2016) |

**Table S1.2**: Sr/Ca ratios of possible sources materials relevant to the southeastern Mediterranean shore.

**References**

Bialik, O. M., & Sisma-Ventura, G. (2016). Proxy-based reconstruction of surface water acidification and carbonate saturation of the Levant Sea during the Anthropocene. *Anthropocene*, In Press. https://doi.org/10.1016/j.ancene.2016.08.001

Krom, M. ., Cliff, R. ., Eijsink, L. ., Herut, B., & Chester, R. (1999). The characterisation of Saharan dusts and Nile particulate matter in surface sediments from the Levantine basin using Sr isotopes. *Marine Geology*, *155*(3–4), 319–330. https://doi.org/10.1016/S0025-3227(98)00130-3

Pastor, F., Valiente, J. A., & Khodayar, S. (2020). A Warming Mediterranean: 38 Years of Increasing Sea Surface Temperature. *Remote Sensing*, *12*(17), 2687. https://doi.org/10.3390/rs12172687

Rahav, E., Paytan, A., Chien, C.-T., Ovadia, G., Katz, T., & Herut, B. (2016). The Impact of Atmospheric Dry Deposition Associated Microbes on the Southeastern Mediterranean Sea Surface Water following an Intense Dust Storm. *Frontiers in Marine Science*, *3*. https://doi.org/10.3389/fmars.2016.00127

Rutten, A., de Lange, G. ., Ziveri, P., Thomson, J., van Santvoort, P. J. ., Colley, S., & Corselli, C. (2000). Recent terrestrial and carbonate fluxes in the pelagic eastern Mediterranean; a comparison between sediment trap and surface sediment. *Palaeogeography, Palaeoclimatology, Palaeoecology*, *158*(3–4), 197–213. https://doi.org/10.1016/S0031-0182(00)00050-X

Sisma-Ventura, G., Bialik, O. M., Yam, R., Herut, B., & Silverman, J. (2017). pCO2 variability in the surface waters of the ultra-oligotrophic Levantine Sea: Exploring the air-sea CO2 fluxes in a fast warming region. *Marine Chemistry*. https://doi.org/10.1016/j.marchem.2017.06.006

Sisma-Ventura, G., Yam, R., Kress, N., & Shemesh, A. (2016). Water column distribution of stable isotopes and carbonate properties in the South-eastern Levantine basin (Eastern Mediterranean): Vertical and temporal change. *Journal of Marine Systems*, *158*, 13–25. https://doi.org/10.1016/j.jmarsys.2016.01.012
