# Supplementary material for: Role of oceanic abiotic carbonate precipitation in future atmospheric CO2 regulation
Source: Sci Rep. 2022 Sep 24;12:15970. doi: 10.1038/s41598-022-20446-7 (PMC9509385; doi:10.1038/s41598-022-20446-7)
Supplement: Supplementary file 2 — Supplementary Information 2. [file 41598_2022_20446_MOESM2_ESM.docx]

Supplement 2 - Materials & Methods

# Study area

The study area is in the south eastern Mediterranean Sea (SEMS), over the inner Israeli shelf (**Figure 1**). This is an ultra-oligotrophic marine environment with exceptionally low nutrient and chlorophyll concentrations. PO_4_^3–^ and NO_3_^-^ concentrations in the surface water range from below detection levels to 0.09 and 0.9 µM respectively and chlorophyll concentrations from 0.003 to 0.415 µg L^–1^ (Herut et al., 2000; Kress et al., 2019); values are higher during autumn and winter compared to the summer. Near surface water temperatures and salinity ranges between 17°C and 38.9‰ in winter and 31°C and 39.8‰ in summer respectively (Ozer et al., 2017). Because of the simple contour of the coastline, the inner Israeli shelf is exposed to winter storms that in rare events (return time of 10–20 years) can reach wave heights of 10m (Katz & Mushkin, 2013). Precipitation in this region is moderate (annual mean ± SD = 497 ± 124 mm; https://ims.data.gov.il) and occurs almost entirely between October and April. During this time, strong rain events can cause sporadic flooding in small coastal rivers that enter the sea (Sandler & Herut, 2000).

## Sea state at the study site

During the sediment trap deployments, we used an up-looking, acoustic Doppler current profiler (ADCP; 600 kHz WH by Teledyne with a temperature sensor), at 26 m depth to measure temperatures near the seafloor and acoustic backscatter intensity (amplitude) in the water column. The ADCP was located approximately 100 m west of the moored traps. Monthly, multiannual (2011-2020) means of temperatures and of salinity at the study area were obtained from CTD sensor (SBE 16 plus by Seabird) measurements from the Israel Oceanographic and Limnological Research (IOLR) monitoring station. This data was collected at the western end of the Hadera power station’s coal pier from 12 m depth.

# Field work

## Sediment traps

Between September 2015 and September 2016, a moored sediment trap setup was deployed ~ 2 km offshore Israel, over the inner continental shelf in the eastern Levantine Basin, Mediterranean Sea (42^o^28’10’’ N; 34^o^51’47’’ E). This setup which was described in detail in Katz and Crouvi (2018) contained a pair of sediment traps deployed in tandem at 15 m depth, 10 m above a 25 m deep, sandy seafloor [(Katz & Crouvi, 2018)](https://www.zotero.org/google-docs/?8EsOyX). The cylindrical traps (section area: 0.015 m^2^, aspect ratio: 4) were placed approximately 100 m to the south of the coal pier of Hadera power station. The traps’ collection bottles which were filled with brine were removed monthly by divers (see deployment periods in **Table 1**). Thereafter, the traps were replaced by new, clean ones with fresh collection bottles. The collection bottles were taken to the laboratory within ~3 h after their removal and placed in the fridge (4^o^C); sample processing commenced within a few (< 4) days.

# Carbon system calculation

Temperature and salinity data from sensors on site (**Figure 2**, main text) allowed the estimation of pCO_2_ and alkalinity, respectively, based on pre-established linear relations in the Levant Surface Waters (LSW) in the region (Sisma-Ventura et al., 2017). Using the Hadera temperature sensor we infer that in early May 2016 the difference between dissolved and atmospheric CO_2_ partial pressure (ΔpCO_2_) increased into the summer. The dissolved CO_2_ levels reached super-saturation by the end of June, where ΔpCO_2_ was over 100 μatm. Variations in alkalinity were much smaller in the scale of 0.04 mEq/L. The temperature sensor was located at 26m, near the seafloor. Considering the gradient of increasing pCO_2_ levels in the upper meters (Sisam-Ventura et al., 2017) and the strong thermal gradient in the region during summer (**Figure 3**), pCO_2_ over saturation is probably somewhat higher near the surface. Ω_aragonite_ values ranged from 3.6 to 4.2 at the depth of the sensors (26m), where values close to 4 appeared in May and increased to a maximum value of 4.2 during the height of summer (August - October). During 2015 there were only 62 days in which Ω_aragonite_ was higher than 4 compared to 109 days in 2016 [Supplument 1, figure S2]. Moreover, the maximum continual period where Ω_aragonite_ was higher than 4 was 90 days in 2016 compared to 30 days in 2015.

*p*CO_2_ can be expressed as a function of temperature at constant DIC and A_T_ [(Takahashi et al., 1993, 2002)](https://www.zotero.org/google-docs/?ya5plX). This temperature dependence of *p*CO_2_ is exceptionally high in the SEMS [(Sisma-Ventura et al., 2016, 2017)](https://www.zotero.org/google-docs/?8HJhDw), where surface water temperatures shift by ~15°C annually, while salinity changes only moderately, between 39.0 PSU and 39.8 PSU [(Bialik & Sisma-Ventura, 2016; Ozer et al., 2017)](https://www.zotero.org/google-docs/?TDAgl7). To obtain the entire set of carbonate properties in sea water, including the CaCO_3_ saturation [(Dickson et al., 2007)](https://www.zotero.org/google-docs/?RWe58m) two of six parameters must be known: TA, DIC, [CO^2-^_3_], [HCO_3_], pH and *p*CO_2_. The *p*CO_2_ values were calculated from the Hadera in-situ temperature sensor, located at depth of 24m, using the *p*CO_2_ temperature (T) dependence equation from Sisma-Ventura et al. (2017) from Eq 1:

(Eq 1) *p*CO_2_ = 14(±0.25)**T*(°C)+113.7(±5.0)

This equation was calibrated for the SEMS, based on multiple research cruises, covering the shelf, slope and the open water environments. High correlation was observed between the thermal and the observed *p*CO_2_ values of the SEMS, with small differences during summer resulting from degassing [(Sisma-Ventura et al., 2017)](https://www.zotero.org/google-docs/?BaUREY). Sea surface total alkalinity is a conservative property in the Mediterranean, and as such, it is strongly correlated with salinity [(Schneider et al., 2007)](https://www.zotero.org/google-docs/?VjXOK9). However, the relation between these two parameters varies significantly on a sub-basin scale [(Cossarini et al., 2015)](https://www.zotero.org/google-docs/?uR2LGN), depending on the regional freshwater balance. We, therefore used the local relationship (Eq 2) to obtain alkalinity values (A_T_) from measured salinities (S) as defined by [Sisma-Ventura et al. (2017)](https://www.zotero.org/google-docs/?1wqHc7):

(Eq 2) *A_T_* = 71.2(±2.3)**S*-162.5(±91.4)

The mean multiannual, monthly salinity data was obtained from the IOLR Hadera station (See results below). Aragonite saturation (Ω_aragonite_) was calculated from the *p*CO_2_ and A_T_ data using the CO2SYS 2.1 program [(Lewis & Wallace, 1998; Excel version)](https://www.zotero.org/google-docs/?tI48jC). Calculations were made using in-situ temperature and salinity, phosphate and silicate of 0.04 and 2.00 μmol kg^-1^ [(Kress et al., 2019)](https://www.zotero.org/google-docs/?b5nqnr), respectively. Carbonate system thermodynamic dissociation constants (K1 and K2) from [Millero (2010)](https://www.zotero.org/google-docs/?q8EH9E), thermodynamic dissociation constants for HSO_4_^-^ (KHSO_4_^-^) from [Dickson (1990)](https://www.zotero.org/google-docs/?L2Dkcb) and the total boron concentration in seawater from [(Lee et al., 2010)](https://www.zotero.org/google-docs/?1yApSv).

The saturation state of a solution with respect to a calcium carbonate was calculated using equation 3:

(Eq 3) Ω = [Ca^2+^][CO_3_^2-^]/K_sp_

where [Ca^2+^] and [CO_3_^2−^] are the *in situ* concentrations in seawater, *K_sp_* is the solubility product of CaCO_3_ (aragonite or calcite) at the *in situ* conditions of temperature, salinity, and pressure. The concentration of Ca^2+^ is assumed to be proportional to the salinity (conservative), and the CO_3_^2−^ is calculated from the selected carbonate properties and the K_1_ and K_2_ of carbonic acid. Ω = 1 suggests a solution in thermodynamic equilibrium with the mineral phase, whereas Ω < 1 or >1 suggests under-saturation and oversaturation, respectively. The released CO_2_ precipitated carbonate ratio (Ψ) was evaluated following (Frankignoulle et al., 1994), mean values calculated for each deployment period and given in Table S2.1. Full calculation of all parameters and error propagations are provided in supplement 4.

| **Month** | **Temperature (°C 11m)** | **Salinity (PSU)** | **A_Crb_ (µmol/kg)** | **Boron (mg/kg)** | **pCO2 (µatm)** | **pH** | **Ψ**  **(mean)** | **Ψ**  **(stdev)** |
| --- | --- | --- | --- | --- | --- | --- | --- | --- |
| **Sep** | 30.00 | 39.91 | 2799.41 | 5.33 | 533.70 | 7.97 | 0.63 | 0.03 |
| **Oct** | 27.00 | 39.76 | 2788.38 | 5.31 | 491.70 | 8.00 | 0.64 | 0.05 |
| **Nov** | 31.00 | 39.75 | 2787.75 | 5.31 | 547.70 | 7.95 | 0.62 | 0.06 |
| **Dec** | 33.00 | 40.23 | 2823.36 | 5.38 | 575.70 | 7.94 | 0.61 | 0.05 |
| **Jan** | 30.00 | 39.33 | 2756.76 | 5.26 | 533.70 | 7.96 | 0.63 | 0.05 |
| **Feb** | 29.00 | 39.32 | 2755.63 | 5.25 | 519.70 | 7.97 | 0.63 | 0.06 |
| **Mar** | 32.00 | 39.28 | 2752.53 | 5.25 | 561.70 | 7.94 | 0.62 | 0.05 |
| **Apr** | 30.00 | 39.30 | 2754.36 | 5.25 | 533.70 | 7.96 | 0.63 | 0.04 |
| **May** | 29.00 | 39.31 | 2755.16 | 5.25 | 519.70 | 7.97 | 0.63 | 0.04 |
| **Jun** | 28.00 | 39.39 | 2760.75 | 5.26 | 505.70 | 7.99 | 0.64 | 0.08 |
| **Jul** | 38.00 | 39.50 | 2769.20 | 5.28 | 645.70 | 7.88 | 0.60 | 0.03 |
| **Aug** | 25.00 | 39.62 | 2778.01 | 5.29 | 463.70 | 8.02 | 0.64 | 0.05 |

**Table S2.1**: Parameters used to calculate Ψ (following Frankignoulle et al., 1994) and values used for calculation of CO_2_ outgassing due to carbonate precipitation. A_Crb_ stands for carbonate alkalinity.

# Sediment traps

Treatment and analysis of material from sediment traps are listed in the following, updated from [Katz and Crouvi (2018)](https://www.zotero.org/google-docs/?k5OlWi) and [Zirks et al. (2021)](https://www.zotero.org/google-docs/?L0wsZG). Methods and references are listed in Table S2.

## Pretreatment sediment trap material

The samples and brine in the collection bottles were transferred into large glass dishes and placed under a stereomicroscope for manual removal of swimmers. The brine and sediment were then transferred to clean pre-weighed, 50 ml falcon tubes and centrifuged at 4000 rpm for 5 min, after which the brine was decanted. To wash away the salts from the sample, distilled water (DW) was added to the falcon tubes at DW/sediment volume ratio of approximately 10, the tubes were agitated with a Vortex-Genie and then centrifuged and decanted as before. This treatment was repeated two more times, thereafter the tubes were placed (cap off) in a drying oven at 60 °C until constant weight was achieved (> 72 h). Samples were then split for various analysis.

## Elements

Strontium (Sr) and calcium (Ca) concentrations in dry sediment from the traps were determined by powdering and homogenizing 2 g subsamples with pastel and mortar and measuring them with an ED-XRF (SPECTROSCOUT) equipped with a vacuum, sampling chamber for improved accuracy. The XRF output was calibrated against concurrent measurements of seven, sediment standards (CRMs). Calibrations for both elements yielded linear correlations with R^2^= 0.99 and the instrument R.S.D (100 x SD/Mean) for repetitive measurements was < 1%. The weight fraction of CaCO_3_ in the sediment from the traps was calculated by multiplying the Ca weight fraction by 2.5.

## Fluxes

After drying, the sediment from each trap was weighed. The total mass flux (TMF) was calculated by dividing the dry sediment weight in each trap by its section area (0.015 m^2^) and by its deployment time (**Table 1**). The TMF mean was of the two sediment traps in the tandem setup and the range (±) was determined as the difference between the flux in each of the traps and their mean. The dry sediment from each collection bottle was first disintegrated and mixed by hand in order to prevent the breakdown of shells and sand particles for PSD microscopic observations, sediment portions for chemical analysis were grinded and homogenized with a pestle and mortar. Fluxes of various constituents in the sediment were calculated by multiplying their measured weight fraction by the TMF.

### Mineralogy

Aragonite and calcite (and other minerals) in sediment trap material were analyzed using a Rigaku MiniFlex benchtop XRD (Cu source, 30 kV/10 mA) between 3° and 70° at 0.05° increments. Some 0.05ml of powdered samples were pressed to a dedicated glass tray and leveled. The mineral abundance of the major phases were determined using the reference intensity ratio method [(Hubbard et al., 1976)](https://www.zotero.org/google-docs/?broken=3CiqoT) using the PDXL Integrated X-ray powder diffraction software with the PDF2 database from the International Centre for Diffraction Data (2016 version), I/I_c_ values of 3.6. 2 and 1 were used for quartz, calcite and aragonite, respectively.

# Supplementary information

To supplement information about the water column we used temperature and salinity data from CTD (Seabird 19 Plus) casts carried out as part of the SoMMoS (Southeastern Mediterranean Monthly cruise Series) project during twelve cruises collected between February 2018 until January 2019 at the edge of the Israeli continental shelf next to the THEMO1 buoy (33.04°N, 034.95°E), see <https://themo.haifa.ac.il/> for more information on the THEMO project. Data is available through the ISRAMAR (<https://isramar.ocean.org.il/isramar2009/>) databases.

# Additional acknowledgments

We thank Barak Herut from the IOLR who headed the “Israel continental shelf research” and obtained the funds for executing the sediment trap research and On Crouvi from the GSI for his willingness to share its data. We are grateful to Elad Israeli, the marine-ops crew of the IOLR and the RV Bat Galim Crew for their dedicated help with the fieldwork. We also thank Yana Yudkovsky and Orit Hyams-Kaphzan for their help with the different laboratory analyses and Lazar Raskin and Tal Ozer from IOLR physical oceanography department for their assistance with obtaining Hadera station CTD and ADCP data. Omri Gadol (University of Haifa) is thanked for graphical assistance. This work used JASMIN, the UK's collaborative data analysis environment (http://jasmin.ac.uk). We also acknowledge the World Climate Research Programme, which, through its Working Group on Coupled Modelling, coordinated and promoted CMIP6. We thank the climate modeling groups for producing and making available their model output, the Earth System Grid Federation (ESGF) for archiving the data and providing access, and the multiple funding agencies who support CMIP6 and ESGF.

# References

Bialik, O.M., Sisma-Ventura, G., 2016. Proxy-based reconstruction of surface water acidification and carbonate saturation of the Levant Sea during the Anthropocene. Anthropocene 16. https://doi.org/10.1016/j.ancene.2016.08.001

Cossarini, G., Lazzari, P., Solidoro, C., 2015. Spatiotemporal variability of alkalinity in the Mediterranean Sea. Biogeosciences 12, 1647–1658. https://doi.org/10.5194/bg-12-1647-2015

Dickson, A.G., 1990. Thermodynamics of the dissociation of boric acid in synthetic seawater from 273 . 15 to 318 . 15 K 37, 755–766.

Dickson, A.G., Sabine, C.L., Christian, J.R., 2007. Guide to Best Practices for Ocean CO2 Measurements. PICES Special Publication 3.

Herut, B., Kress, N., Hornung, H., 2000. Nutrient pollution at the lower reaches of Mediterranean coastal rivers in Israel. Water Sci. Technol. 42, 147–152. https://doi.org/10.2166/wst.2000.0306

Hubbard, C.R., Evans, E.H., Smith, D.K., 1976. The reference intensity ratio, I / I c , for computer simulated powder patterns. J. Appl. Crystallogr. 9, 169–174. https://doi.org/10.1107/s0021889876010807

Katz, O., Mushkin, A., 2013. Characteristics of sea-cliff erosion induced by a strong winter storm in the eastern Mediterranean. Quat. Res. 80, 20–32. https://doi.org/10.1016/j.yqres.2013.04.004

Katz, T., Crouvi, O., 2018. Sediment flux dynamics over the shallow (25 m depth) shelf of the Mediterranean Sea along the Israeli coast. Mar. Geol. 406, 1–11. https://doi.org/10.1016/j.margeo.2018.09.004

Kress, N., Rahav, E., Silverman, J., Herut, B., 2019. Environmental status of Israel’s Mediterranean coastal waters: Setting reference conditions and thresholds for nutrients, chlorophyll-a and suspended particulate matter. Mar. Pollut. Bull. 141, 612–620. https://doi.org/10.1016/j.marpolbul.2019.02.070

Lee, K., Kim, T.-W., Byrne, R.H., Millero, F.J., Feely, R.A., Liu, Y.-M., 2010. The universal ratio of boron to chlorinity for the North Pacific and North Atlantic oceans. Geochim. Cosmochim. Acta 74, 1801–1811. https://doi.org/10.1016/j.gca.2009.12.027

Lewis, E.R., Wallace, D.W.R., 1998. Program Developed for CO2 System Calculations. Environmental System Science Data Infrastructure for a Virtual Ecosystem, cdiac:CDIAC-105. https://doi.org/10.15485/1464255

Millero, F.J., 2010. Carbonate constants for estuarine waters. Mar. Freshw. Res. 61, 139. https://doi.org/10.1071/MF09254

Ozer, T., Gertman, I., Kress, N., Silverman, J., Herut, B., 2017. Interannual thermohaline (1979–2014) and nutrient (2002–2014) dynamics in the Levantine surface and intermediate water masses, SE Mediterranean Sea. Glob. Planet. Change 151, 60–67. https://doi.org/10.1016/j.gloplacha.2016.04.001

Sandler, A., Herut, B., 2000. Composition of clays along the continental shelf off Israel: contribution of the Nile versus local sources. Mar. Geol. 167, 339–354. https://doi.org/10.1016/S0025-3227(00)00021-9

Schneider, A., Wallace, D.W.R., Körtzinger, A., 2007. Alkalinity of the Mediterranean Sea. Geophys. Res. Lett. 34. https://doi.org/10.1029/2006GL028842

Sisma-Ventura, G., Bialik, O.M., Yam, R., Herut, B., Silverman, J., 2017. pCO<inf>2</inf> variability in the surface waters of the ultra-oligotrophic Levantine Sea: Exploring the air-sea CO<inf>2</inf> fluxes in a fast warming region. Mar. Chem. https://doi.org/10.1016/j.marchem.2017.06.006

Sisma-Ventura, G., Yam, R., Kress, N., Shemesh, A., 2016. Water column distribution of stable isotopes and carbonate properties in the South-eastern Levantine basin (Eastern Mediterranean): Vertical and temporal change. J. Mar. Syst. 158, 13–25. https://doi.org/10.1016/j.jmarsys.2016.01.012

Frankignoulle, M., Canon, C., Gattuso, J.-P., 1994. Marine calcification as a source of carbon dioxide: Positive feedback of increasing atmospheric CO 2. Limnol. Oceanogr. 39, 458–462. https://doi.org/10.4319/lo.1994.39.2.0458

Zirks, E., Krom, M., Schmiedl, G., Katz, T., Xiong, Y., Alcott, L.J., Poulton, S.W., Goodman-Tchernov, B., 2021. Redox evolution and the development of oxygen minimum zones in the Eastern Mediterranean Levantine basin during the early Holocene. Geochim. Cosmochim. Acta 297, 82–100. https://doi.org/10.1016/j.gca.2021.01.009

| **Analysis** | **Method** | **Instrument** | **Described in** |
| --- | --- | --- | --- |
| Pretreatment of sediment trap material | Swimmers and salts removal and drying. | Standard lab equipment | Katz & Crouvi (2018) |
| Total mass flux | Gravimetric | Standard lab equipment | Katz & Crouvi (2018) |
| Elemental analysis (Ca, Sr) | Elements by X-Ray Fluorescence | SPECTROSCOUT  -Geo | Zirks et al., (2021) |

**Table 2S**: summary of previously published methods and treatment used on the sediment trap samples. Full description and details are given in the above.
