# Supplementary material for: Role of oceanic abiotic carbonate precipitation in future atmospheric CO2 regulation
Source: Sci Rep. 2022 Sep 24;12:15970. doi: 10.1038/s41598-022-20446-7 (PMC9509385; doi:10.1038/s41598-022-20446-7)
Supplement: Supplementary file 3 — Supplementary Information 3. [file 41598_2022_20446_MOESM3_ESM.docx]

Supplement 3: Evidence from CMIP6

To gauge how rates of inorganic aragonite precipitation may change in response to climate change over the coming century, we have supplemented our observations from the eastern Mediterranean with biogeochemical hindcasts and forecasts from the Coupled Model Intercomparison Project Phase 6, specifically (CMIP6 Eyring et al., 2016; Orr et al., 2017). The historical (1850-2014) experiment is used for validation purposes, and the SSP2-4.5 and SSP5-8.5 experiments (2015-2100) from ScenarioMIP are used as mid-range and pessimistic estimates of the 21^st^ Century climate trajectory (Gidden et al., 2019; O’Neill et al., 2017, 2016).

All data sets used for this supplement are made available at (Vogt-Vincent et al., 2021). The scripts used in this section are also available at (Vogt-Vincent et al., 2021), as well as the following github repository: https://github.com/nvogtvincent/med_aragonite.

##### Data sources

Monthly mean sea-surface temperature (SST) and the required variables to calculate sea-surface $\Omega_{Aragonite}$ were available for three Earth System Models (ESMs) on JASMIN (Lawrence et al., 2013): UKESM1.0-LL (Sellar et al., 2019), GFDL-CM4 (Held et al., 2019), and CanESM5 (Swart et al., 2019). All variables were bilinearly interpolated onto a regular 1^o^ grid. A full list of variables used is given in Table S3.1.

For validation purposes, CMIP6 historical sea-surface pH, TA and SST were compared against observations and reanalyses from the eastern Mediterranean. Unfortunately, pH was not available for CanESM5. Historical pH and TA records were obtained from in-situ direct and proxy measurements (Bialik and Sisma-Ventura, 2016; Sisma-Ventura et al., 2017, 2016), and SST was obtained from the ERA5 reanalysis (Hersbach et al., 2020).

##### Inorganic aragonite precipitation rate parameterisation

To estimate changes in the rate of inorganic aragonite precipitation, $R_{ai}$, we derived an inorganic aragonite precipitation rate parameterisation by assuming the precipitation rate follows a power-law dependence on the aragonite saturation state:

$$R_{ai}(T,\Omega)=k\left( T \right)\left[ \Omega-1 \right]^{n(T)}$$

where $T$ is the precipitation temperature (SST), $\Omega$ is the aragonite saturation state ($\Omega_{aragonite}$), and $k(T)$ and $n(T)$ are respectively the reaction rate constants and order, both of which are a function of temperature. Equations for $k(T)$ and $n(T)$ were obtained by fitting second and first order polynomials to the estimates of $k$ and $T$ from Burton & Walter (1987), which were measured at 5, 25 and 37^o^C.

The resulting parameterisation is:

$$R_{ai}(T,\Omega)=k\left( T \right)\left[ \Omega-1 \right]^{n(T)}$$

$$k\left( T \right)=0.357+0.0353T-0.000424T^{2}$$

$$n\left( T \right)=0.0985+0.0628T$$

This parameterisation was calculated with **aragonite_parameters.py** and is visualised in figure S3.1.

##### Data processing

###### $\Omega_{aragonite}$ calculation

$\Omega_{Aragonite}$ was calculated from surface Total Alkalinity (TA) and pCO_2_ with PyCO2SYS (Humphreys et al., 2020; Lewis and Wallace, 1998), adjusted for in-situ salinity and temperature, and using equilibrium constant parameterisations from Sulpis et al. (2020). Nutrient concentrations were assumed to be zero as these variables were not consistently available from the ESMs used, and a sensitivity analysis based on output from UKESM1.0-LL indicated that the calculated $\Omega_{Aragonite}$ was relatively insensitive to these values outside of the polar seas. The relevant script is **calculate_aragonite.py**.

###### Validation

For comparison against observations and reanalyses for validation purposes, monthly mean gridded variables (from CMIP6 and ERA5) were averaged across the eastern Mediterranean (east of 32^o^E). Although it would have been possible to simply extract data from the grid cell/s corresponding to the observations, CMIP6 model resolution is coarse relative to the dimensions of the eastern Mediterranean so this average was taken to minimise the effects of cell-level noise. The relevant script is **model_validation.py**.

###### $R_{ai}$ (inorganic aragonite precipitation rate)

For each model, monthly mean $R_{ai}$ was calculated as a gridded variable with the previously described $R_{ai}(T, \Omega)$ parameterisation using CMIP SST and surface $\Omega_{aragonite}$. Projections for how $R_{ai}$ may change in the eastern Mediterranean in the future were generated by averaging monthly mean $R_{ai}$ across the eastern Mediterranean (east of 32^o^E).

Additional ‘SST-only’ and ‘$\Omega$-only’ scenarios for $R_{ai}$ were generated to decouple the effects of rising SST and falling $\Omega_{aragonite}$. For the SST-only scenario, monthly mean $R_{ai}$ was calculated using the monthly *climatological* value of $\Omega_{aragonite}$ from 1850-1899, and monthly mean SST. For the $\Omega$-only scenario, monthly mean $R_{ai}$ was calculated using the monthly *climatological* value of SST from 1850-1900, and monthly mean $\Omega_{aragonite}$. Note that since $R_{ai}$ is nonlinear, ${\Delta R}_{ai}\neq\Delta R_{ai}^{SST-only}+\Delta R_{ai}^{\Omega-only}$. The relevant scripts are **calculate_aragonite_monclim.py** (to calculate the 1850-1899 monthly climatologies) and **precipitation_rate_time_series.py** (to plot $R_{ai}$, $R_{ai}^{SST-only}$ and $R_{ai}^{\Omega-only}$ for a region of interest).


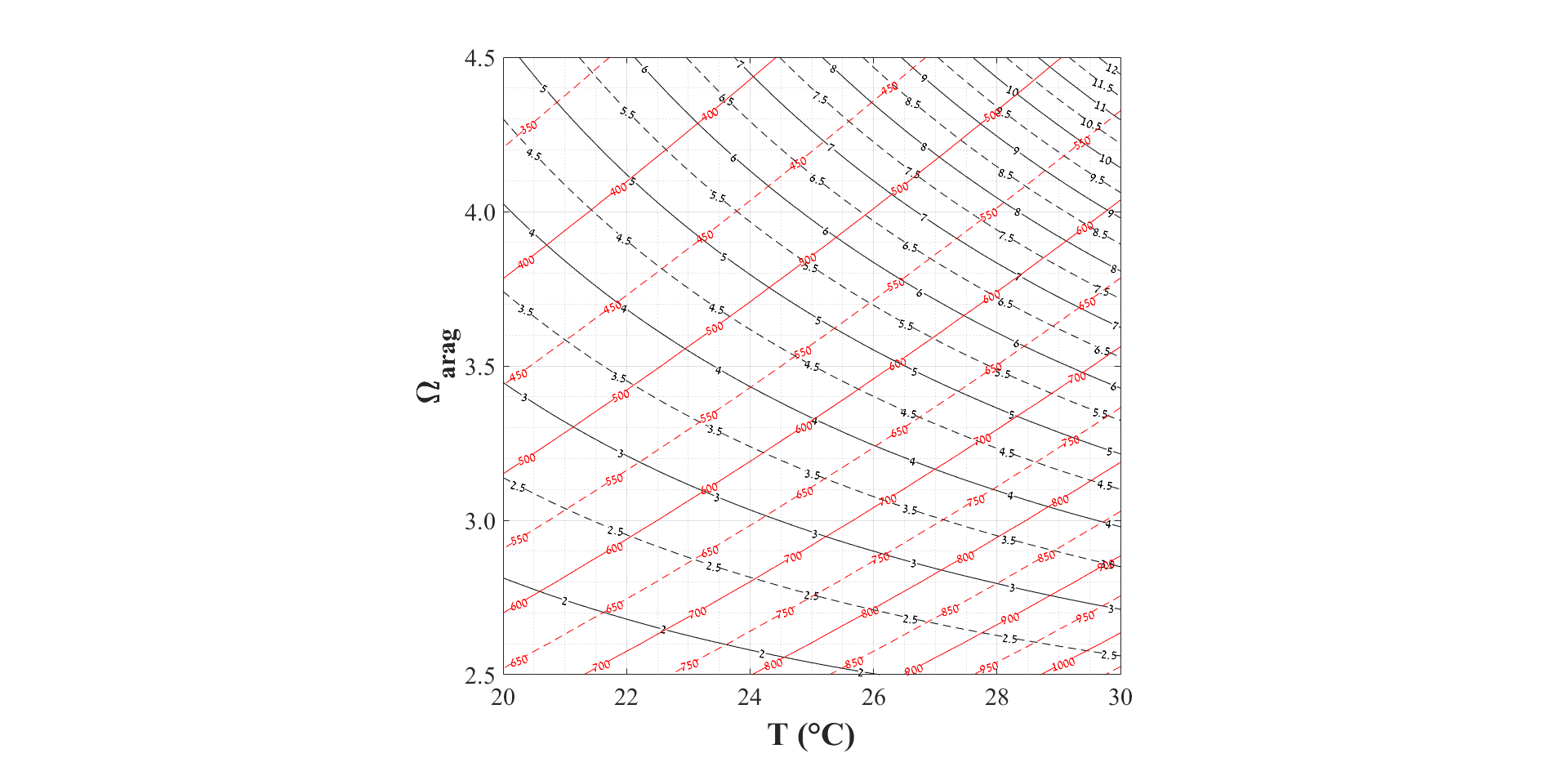


Figure S3.1: Inorganic aragonite precipitation rate as a function of temperature and $\Omega_{aragonite}$ following the parameterisation described in this section, derived from the results of Burton & Walter (1987). Red lines represent trends for a given pCO2 and black lines are trends for a given pH.

##### CMIP6 Model Validation

CMIP6 pH, TA and SST are compared against direct and proxy observations (pH and TA) and a reanalysis (SST) in Figure S3.1. Both CMIP6 models with pH variables (UKESM1-0-LL and GFDL-CM4) reproduce the observed decline in pH over the latter half of the 20^th^ Century well, although the absolute value may be slightly too high. For TA, model-observation agreement is good for GFDL-CM4 and CanESM5 but is poorer for UKESM1-0-LL, which may be due to a very large TA anomaly simulated by the model around the mouth of the Nile River. All CMIP6 models reproduce very similar SST trends for the 20^th^ Century, and this agrees well with the ERA5 reanalysis. In summary, aside from a questionable model-observation offset in TA for UKESM1-0-LL, all CMIP6 models do reasonably well at reproducing observed trends in the physical and biogeochemical variables relevant to inorganic aragonite precipitation for the latter half of the 20^th^ Century, which may provide some degree of confidence in predictions for 21^st^ Century climate trajectories.


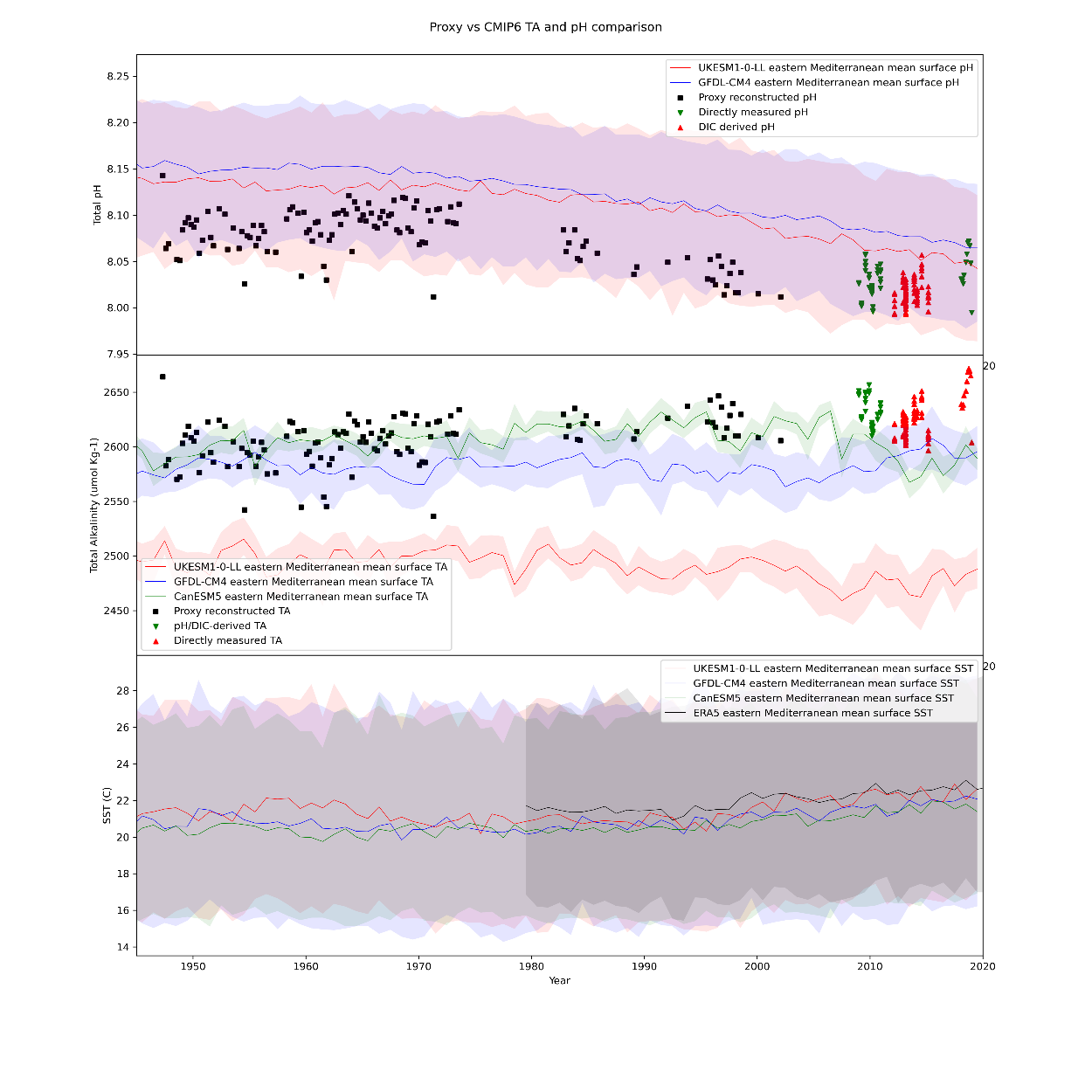


Figure S3.2: **Top panel**: Eastern Mediterranean sea-surface pH from CMIP6 models (blue and red lines) against observed and proxy pH from eastern Mediterranean sites (markers). For the CMIP6 pH, the solid line represents the annual mean (of monthly mean pH averaged across the eastern Mediterranean) and the shaded region covers the annual range (of monthly mean pH averaged across the eastern Mediterranean). Note that pH was not available for CanESM5. **Central panel:**Eastern Mediterranean sea-surface Total Alkalinity (TA) from CMIP6 models (blue, red and green lines) against observed and proxy TA from eastern Mediterranean sites (markers).  **Bottom panel:**Eastern Mediterranean sea-surface temperature (SST) from CMIP6 models (blue, red and green lines) against eastern Mediterranean SST from ERA5 (black line). The CMIP6 output used in these figures is from the historical (-2014) and SSP2-4.5 (2015-) scenarios.

##### Aragonite precipitation trends in the eastern Mediterranean

The estimated inorganic aragonite precipitation rate for the eastern Mediterranean, $R_{ai}$, as well as the $R_{ai}^{SST-only}$ and $R_{ai}^{\Omega-only}$ scenarios to decouple the effects of SST and $\Omega_{aragonite}$, is shown in Figure 5 (also here as S3.3) for the three CMIP6 models used in this study. Although there is some variation amongst the models in the magnitude of inorganic aragonite precipitation rate predicted, all three models predict a reduction in precipitation rate of around 30-40% in the eastern Mediterranean by the end of the 21^st^ Century relative to the 20^th^ Century for the moderate SSP2-4.5 climate trajectory. Whilst the warming predicted for the eastern Mediterranean pushes the precipitation rate towards higher values (red lines), this is insufficient to counteract the much stronger effect predicted as a result of the reduction in $\Omega_{aragonite}$ (blue lines).

Indeed, this predicted trend is mirrored by the global ocean (figure S3.4). With this parameterisation, even under the moderate SSP2-4.5 climate trajectory, the predicted reduction in $\Omega_{aragonite}$ ‘outpaces’ the increase in SST, resulting in a reduction in $R_{ai}$ practically everywhere.

##### Discussion

The trends in $\Omega_{aragonite}$ and SST predicted by the CMIP6 models used in this study for the eastern Mediterranean as described above are robust and generally agree well with observations for the historical period. The *key uncertainty* when translating these trends into quantitative (or even qualitative) predictions for inorganic aragonite precipitation rate is the parameterisation used in this study.

The parameterised function $R_{ai}(T,\Omega)$ is has low sensitivity to the uncertainty in $k$ and $n$ reported in Burton & Walter (1987). However, since this parameterisation was effectively derived from three reported sets of values of $k$ and $n$, the true uncertainty will be significantly higher. Additionally, the experiments of Burton & Walter (1987) were performed in laboratory conditions and were ‘seeded’, with the results normalised by the seed surface area. Any use of this $R_{ai}(T,\Omega)$ parameterisation therefore relies on the following assumptions:

- The surface area available for heterogeneous nucleation is constant in space and time (if $R$ is proportional to seeded surface area)
- The functions $k(T)$ and $n(T)$ are (i) smooth and (ii) not fundamentally different in ‘real’ oceanic conditions compared to laboratory conditions

The first assumption is very likely incorrect, but at present there is insufficient data to quantify ‘how incorrect’ it is. We therefore proceed with these assumptions, acknowledging that improved observational constraints on the controls of inorganic aragonite precipitation rate are urgently needed.

##### Data availability

Scripts, results and additional material will be made available via a the Figshare repository at 10.6084/m9.figshare.15121131.


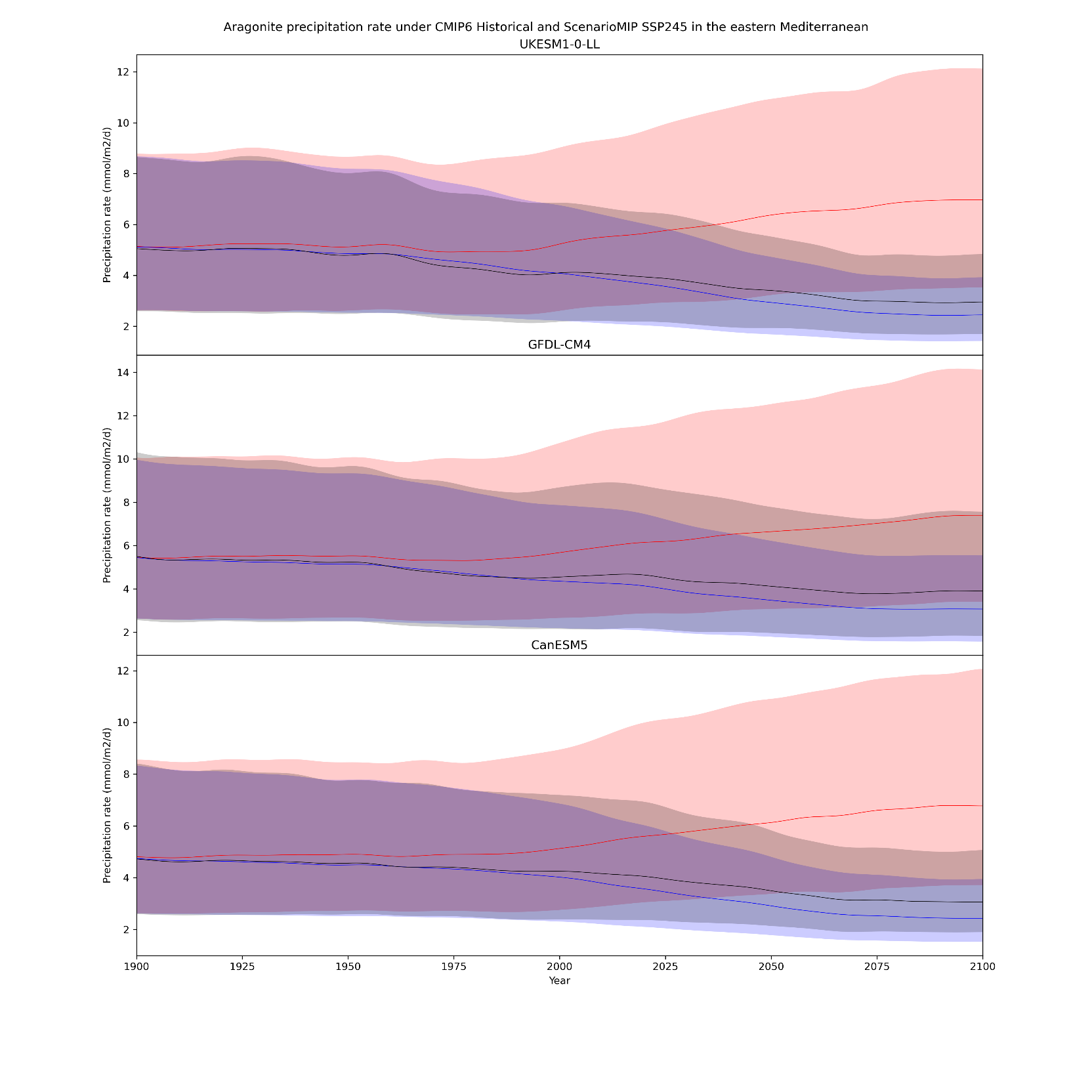


Figure S3.3: $R_{ai}$ (black line), $R_{ai}^{SST-only}$(red line) and $R_{ai}^{\Omega-only}$(blue line) from CMIP6 Historical and ScenarioMIP SSP2-4.5 for the eastern Mediterranean. The $R_{ai}$ scenario reflects the ‘true’ CMIP prediction, whereas the $R_{ai}^{SST-only}$ and $R_{ai}^{\Omega-only}$scenarios fix $\Omega_{aragonite}$ and SST respectively to 1850-1899 climatological values. The top, central and bottom panels are UKESM1-0-LL, GFDL-CM4, and CanESM5 respectively. As in figure S3.2, the line represents the annual mean (of monthly mean $R_{ai}$ calculated across the eastern Mediterranean) and the shaded region spans the annual range. All time series were passed through a gaussian filter with $\sigma=5$ years to make the plot clearer. It is important to reiterate that the ‘m^2^’ in the precipitation rate units refers to the surface area of seeds available for heterogeneous nucleation, not ocean surface area.


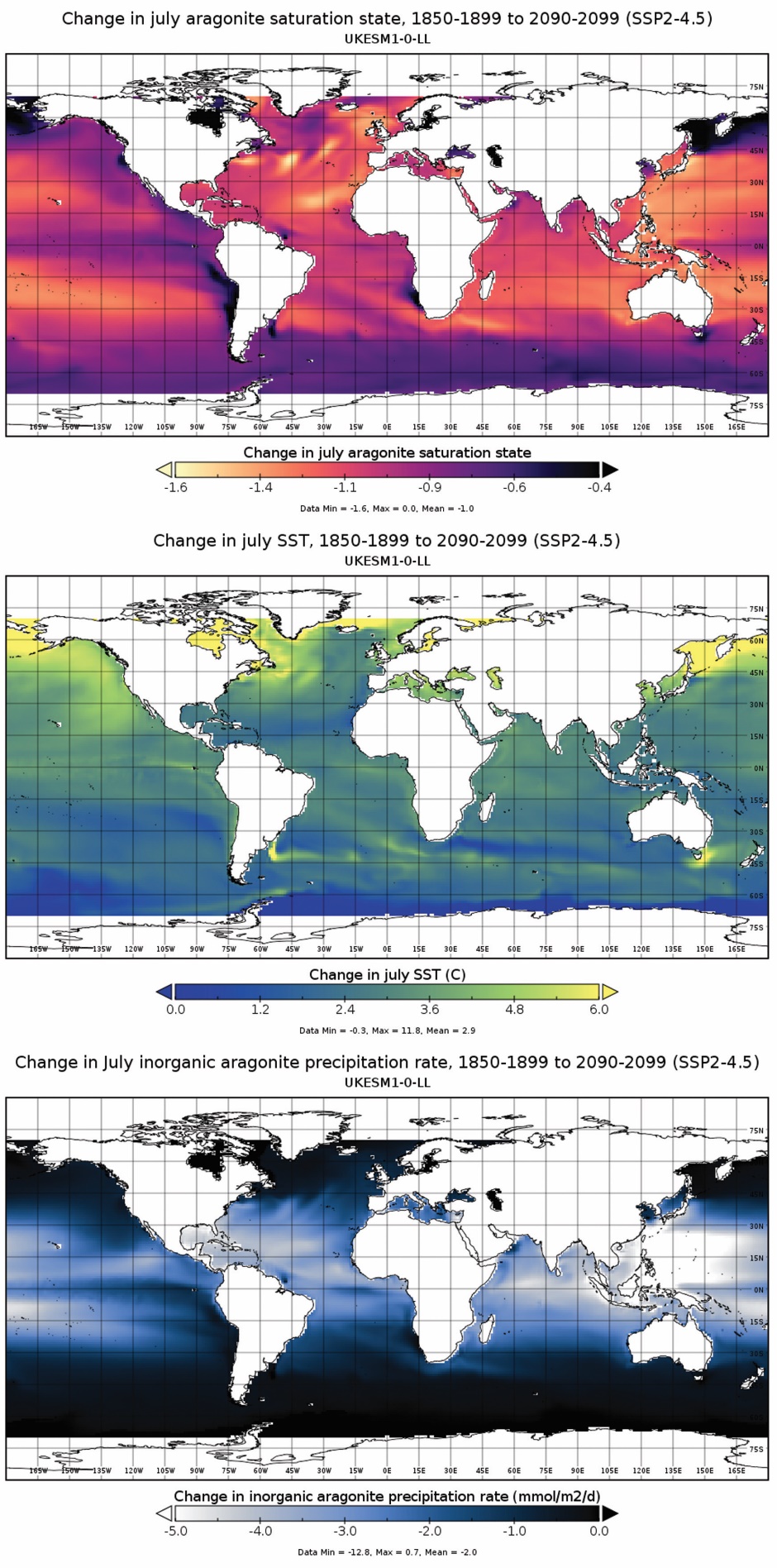


Figure S3.4: Global changes in July $\Omega_{aragonite}$ (top panel), SST (central panel), and $R_{ai}$ (bottom panel) predicted by UKESM1-0-LL under the moderate SSP2-4.5 climate trajectory from 1850-1899 (late 19^th^ Century) to 2090-2099 (late 21^st^ Century).

| **Institution** | **Model** | **Experiment** | **Variable** | **Version** |
| --- | --- | --- | --- | --- |
| MOHC | UKESM1-0-LL | CMIP Historical | phos | 20210508 |
|  |  |  | talk | 20190624 |
|  |  |  | tos | 20190624 |
|  |  |  | sos | 20190624 |
|  |  |  | spco2 | 20190624 |
|  |  | ScenarioMIP SSP2-4.5 | phos | 20210427 |
|  |  |  | talk | 20190708 |
|  |  |  | tos | 20190418 |
|  |  |  | sos | 20190418 |
|  |  |  | spco2 | 20190708 |
|  |  | ScenarioMIP SSP5-8.5 | phos | 20210412 |
|  |  |  | talk | 20210412 |
|  |  |  | tos | 20190724 |
|  |  |  | sos | 20190724 |
|  |  |  | spco2 | 20190724 |
| NOAA-GFDL | GFDL-CM4 | CMIP Historical | ph | 20190307 |
|  |  |  | talk | 20190307 |
|  |  |  | tos | 20190223 |
|  |  |  | sos | 20190223 |
|  |  |  | spco2 | 20190223 |
|  |  | ScenarioMIP SSP2-4.5 | ph | 20190317 |
|  |  |  | talk | 20190317 |
|  |  |  | tos | 20190317 |
|  |  |  | sos | 20190317 |
|  |  |  | spco2 | 20190317 |
|  |  | ScenarioMIP SSP5-8.5 | ph | 20190318 |
|  |  |  | talk | 20190318 |
|  |  |  | tos | 20190318 |
|  |  |  | sos | 20190318 |
|  |  |  | spco2 | 20190318 |
| CCCma | CanESM5 | CMIP6 Historical | talk | 20190501 |
|  |  |  | tos | 20190501 |
|  |  |  | sos | 20190501 |
|  |  |  | spco2 | 20190501 |
|  |  | ScenarioMIP SSP2-4.5 | talk | 20190502 |
|  |  |  | tos | 20190502 |
|  |  |  | sos | 20190502 |
|  |  |  | spco2 | 20190502 |
|  |  | ScenarioMIP SSP5-8.5 | talk | 20190502 |
|  |  |  | tos | 20190502 |
|  |  |  | sos | 20190502 |
|  |  |  | spco2 | 20190502 |

Table S3.1: All CMIP6 variables used in this study

##### References for Supplementary Materials 3

Bialik, O.M., Sisma-Ventura, G., 2016. Proxy-based reconstruction of surface water acidification and carbonate saturation of the Levant Sea during the Anthropocene. Anthropocene In Press. https://doi.org/10.1016/j.ancene.2016.08.001

Burton, E.A., Walter, L.M., 1987. Relative precipitation rates of aragonite and Mg calcite from seawater: Temperature or carbonate ion control? Geology 15, 111. https://doi.org/10.1130/0091-7613(1987)15<111:RPROAA>2.0.CO;2

Eyring, V., Bony, S., Meehl, G.A., Senior, C.A., Stevens, B., Stouffer, R.J., Taylor, K.E., 2016. Overview of the Coupled Model Intercomparison Project Phase 6 (CMIP6) experimental design and organization. Geosci. Model Dev. 9, 1937–1958. https://doi.org/10.5194/gmd-9-1937-2016

Gidden, M.J., Riahi, K., Smith, S.J., Fujimori, S., Luderer, G., Kriegler, E., Van Vuuren, D.P., Van Den Berg, M., Feng, L., Klein, D., Calvin, K., Doelman, J.C., Frank, S., Fricko, O., Harmsen, M., Hasegawa, T., Havlik, P., Hilaire, J., Hoesly, R., Horing, J., Popp, A., Stehfest, E., Takahashi, K., 2019. Global emissions pathways under different socioeconomic scenarios for use in CMIP6: A dataset of harmonized emissions trajectories through the end of the century. Geosci. Model Dev. 12, 1443–1475. https://doi.org/10.5194/gmd-12-1443-2019

Held, I.M., Guo, H., Adcroft, A., Dunne, J.P., Horowitz, L.W., Krasting, J., Shevliakova, E., Winton, M., Zhao, M., Bushuk, M., Wittenberg, A.T., Wyman, B., Xiang, B., Zhang, R., Anderson, W., Balaji, V., Donner, L., Dunne, K., Durachta, J., Gauthier, P.P.G., Ginoux, P., Golaz, J.C., Griffies, S.M., Hallberg, R., Harris, L., Harrison, M., Hurlin, W., John, J., Lin, P., Lin, S.J., Malyshev, S., Menzel, R., Milly, P.C.D., Ming, Y., Naik, V., Paynter, D., Paulot, F., Rammaswamy, V., Reichl, B., Robinson, T., Rosati, A., Seman, C., Silvers, L.G., Underwood, S., Zadeh, N., 2019. Structure and Performance of GFDL’s CM4.0 Climate Model. J. Adv. Model. Earth Syst. 11, 3691–3727. https://doi.org/10.1029/2019MS001829

Hersbach, H., Bell, B., Berrisford, P., Hirahara, S., Horányi, A., Muñoz-Sabater, J., Nicolas, J., Peubey, C., Radu, R., Schepers, D., Simmons, A., Soci, C., Abdalla, S., Abellan, X., Balsamo, G., Bechtold, P., Biavati, G., Bidlot, J., Bonavita, M., De Chiara, G., Dahlgren, P., Dee, D., Diamantakis, M., Dragani, R., Flemming, J., Forbes, R., Fuentes, M., Geer, A., Haimberger, L., Healy, S., Hogan, R.J., Hólm, E., Janisková, M., Keeley, S., Laloyaux, P., Lopez, P., Lupu, C., Radnoti, G., de Rosnay, P., Rozum, I., Vamborg, F., Villaume, S., Thépaut, J.N., 2020. The ERA5 global reanalysis. Q. J. R. Meteorol. Soc. 146, 1999–2049. https://doi.org/10.1002/qj.3803

Humphreys, M.P., Gregor, L., Pierrot, D., van Heuven, S., Lewis, E.R., Wallace, D.W.R., 2020. PyCO2SYS: marine carbonate system calculations in Python. https://doi.org/10.5281/ZENODO.3967359

Lawrence, B.N., Bennett, V.L., Churchill, J., Juckes, M., Kershaw, P., Pascoe, S., Pepler, S., Pritchard, M., Stephens, A., 2013. Storing and manipulating environmental big data with JASMIN. Proc. - 2013 IEEE Int. Conf. Big Data, Big Data 2013 68–75. https://doi.org/10.1109/BigData.2013.6691556

Lewis, E.R., Wallace, D.W.R., 1998. Program Developed for CO2 System Calculations. Environmental System Science Data Infrastructure for a Virtual Ecosystem, cdiac:CDIAC-105. https://doi.org/10.15485/1464255

O’Neill, B.C., Kriegler, E., Ebi, K.L., Kemp-Benedict, E., Riahi, K., Rothman, D.S., van Ruijven, B.J., van Vuuren, D.P., Birkmann, J., Kok, K., Levy, M., Solecki, W., 2017. The roads ahead: Narratives for shared socioeconomic pathways describing world futures in the 21st century. Glob. Environ. Chang. 42, 169–180. https://doi.org/10.1016/j.gloenvcha.2015.01.004

O’Neill, B.C., Tebaldi, C., Van Vuuren, D.P., Eyring, V., Friedlingstein, P., Hurtt, G., Knutti, R., Kriegler, E., Lamarque, J.F., Lowe, J., Meehl, G.A., Moss, R., Riahi, K., Sanderson, B.M., 2016. The Scenario Model Intercomparison Project (ScenarioMIP) for CMIP6. Geosci. Model Dev. 9, 3461–3482. https://doi.org/10.5194/gmd-9-3461-2016

Orr, J.C., Najjar, R.G., Aumont, O., Bopp, L., Bullister, J.L., Danabasoglu, G., Doney, S.C., Dunne, J.P., Dutay, J.C., Graven, H., Griffies, S.M., John, J.G., Joos, F., Levin, I., Lindsay, K., Matear, R.J., McKinley, G.A., Mouchet, A., Oschlies, A., Romanou, A., Schlitzer, R., Tagliabue, A., Tanhua, T., Yool, A., 2017. Biogeochemical protocols and diagnostics for the CMIP6 Ocean Model Intercomparison Project (OMIP). Geosci. Model Dev. 10, 2169–2199. https://doi.org/10.5194/gmd-10-2169-2017

Sellar, A.A., Jones, C.G., Mulcahy, J.P., Tang, Y., Yool, A., Wiltshire, A., O’Connor, F.M., Stringer, M., Hill, R., Palmieri, J., Woodward, S., de Mora, L., Kuhlbrodt, T., Rumbold, S.T., Kelley, D.I., Ellis, R., Johnson, C.E., Walton, J., Abraham, N.L., Andrews, M.B., Andrews, T., Archibald, A.T., Berthou, S., Burke, E., Blockley, E., Carslaw, K., Dalvi, M., Edwards, J., Folberth, G.A., Gedney, N., Griffiths, P.T., Harper, A.B., Hendry, M.A., Hewitt, A.J., Johnson, B., Jones, A., Jones, C.D., Keeble, J., Liddicoat, S., Morgenstern, O., Parker, R.J., Predoi, V., Robertson, E., Siahaan, A., Smith, R.S., Swaminathan, R., Woodhouse, M.T., Zeng, G., Zerroukat, M., 2019. UKESM1: Description and Evaluation of the U.K. Earth System Model. J. Adv. Model. Earth Syst. 11, 4513–4558. https://doi.org/10.1029/2019MS001739

Sisma-Ventura, G., Bialik, O.M., Yam, R., Herut, B., Silverman, J., 2017. pCO2 variability in the surface waters of the ultra-oligotrophic Levantine Sea: Exploring the air-sea CO2 fluxes in a fast warming region. Mar. Chem. https://doi.org/10.1016/j.marchem.2017.06.006

Sisma-Ventura, G., Yam, R., Kress, N., Shemesh, A., 2016. Water column distribution of stable isotopes and carbonate properties in the South-eastern Levantine basin (Eastern Mediterranean): Vertical and temporal change. J. Mar. Syst. 158, 13–25. https://doi.org/10.1016/j.jmarsys.2016.01.012

Sulpis, O., Lauvset, S.K., Hagens, M., 2020. Current estimates of K1∗and K2∗appear inconsistent with measured CO2 system parameters in cold oceanic regions. Ocean Sci. 16, 847–862. https://doi.org/10.5194/os-16-847-2020

Swart, N.C., Cole, J.N.S., Kharin, V. V., Lazare, M., Scinocca, J.F., Gillett, N.P., Anstey, J., Arora, V., Christian, J.R., Hanna, S., Jiao, Y., Lee, W.G., Majaess, F., Saenko, O.A., Seiler, C., Seinen, C., Shao, A., Sigmond, M., Solheim, L., Von Salzen, K., Yang, D., Winter, B., 2019. The Canadian Earth System Model version 5 (CanESM5.0.3). Geosci. Model Dev. 12, 4823–4873. https://doi.org/10.5194/gmd-12-4823-2019

Vogt-Vincent, N., Bialik, O., Sisma-Ventura, G., Silverman, J., Katz, T., 2021. CMIP6 data and scripts for abiotic aragonite precipitation in the eastern Mediterranean. Figshare. https://doi.org/10.6084/m9.figshare.15121131.v1
